# Advances in the total synthesis of bis- and tris-indole alkaloids containing N-heterocyclic linker moieties

**DOI:** 10.1039/d4np00012a

**Published:** 2024-04-26

**Authors:** Kyra R. Dvorak, Jetze J. Tepe

**Affiliations:** a Michigan State University USA dvorakky@msu.edu; b University of Virginia USA

## Abstract

The past several years have seen an increase in the discovery and isolation of natural products of the indole alkaloid class. Bis- and tris-indole alkaloids are classes of natural products that have been shown to display diverse, potent biological activities. Of particular interest are bis- and tris-indole alkaloids containing N-heterocyclic linker moieties. It has been reported that more than 85% of biologically active compounds contain one or more heterocyclic moieties; of these, N-heterocycles have been identified as the most prevalent. The goal of this review is to provide a detailed overview of the recent advances in isolation and total synthesis of bis- and tris-indole alkaloids that contain N-heterocyclic linker moieties. The known biological activities of these natural products will also be discussed.

## Abstract 2

This review highlights advances in isolation and synthesis of bis- and tris-indole alkaloids containing *N*-heterocyclic linker moieties. Natural products in this class are of particular significance due to their potent biological activities.

## Graphical abstract

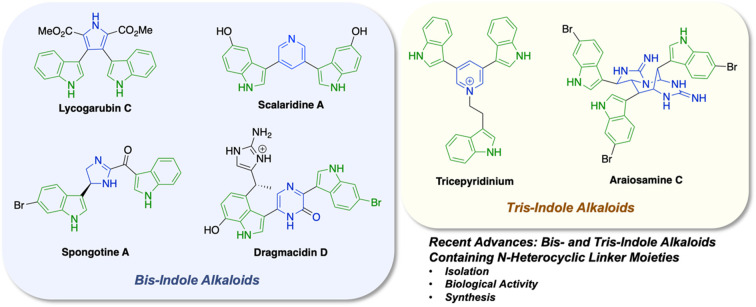

## 1. Introduction

As their name suggests, indole alkaloids are natural products that contain one or more indole structural moieties. They are commonly isolated from a variety of marine sources, including sponges, tunicates, red algae, acorn worms, and symbiotic bacteria, and they represent the largest, and among the most complicated, class of the marine alkaloids.^1^ Indole alkaloids have been shown to display diverse biological activities, including cytotoxic, antitumor, antiviral, antibacterial, and anti-inflammatory activities.^2^ The structure, activity, and synthesis of indole alkaloids have been discussed in several reviews over the years.^3^ The goal of this review is to provide a detailed overview on recent advances in isolation and total synthesis of bis- and tris-indole alkaloid natural products that contain N-heterocyclic linker moieties. These heterocyclic moieties are of particular interest as it has been reported that more than 85% of biologically active compounds contain one or more heterocyclic moieties with N-heterocycles being the most prevalent.^4^ These heterocyclic moieties are of particular interest as it has been reported that more than 85% of biologically active compounds contain one or more heterocyclic moieties.^5^ The biological activities of these natural products will also be discussed.

## 2. Bis-indole alkaloids

### 2.1 Imidazole and imidazoline linker moieties

#### 2.1.1. Topsentins and Spongotines

**Fig. 1:**
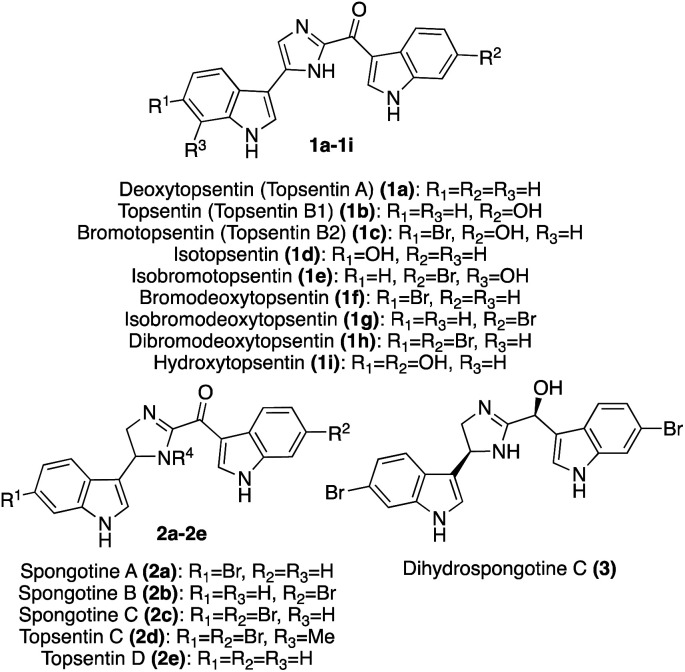
Chemical structures of Topsentin and Spongotine natural products (1a–h, 2a–e, 3) and the synthetic Topsentin analogue Hydroxytopsentin (1i).

Topsentins and Spongotines are classes of marine alkaloid bis-indole natural products that contain characteristic 2-carbonylimidazole or 2-carbonylimidazoline linker moieties between their two indole fragments. The first few natural products that were discovered in these classes were Deoxytopsentin (Topsentin A) (1a), Topsentin (Topsentin B1) (1b), and Bromotopsentin (Topsentin B2) (1c). These three natural products were first isolated from Mediterranean marine sponge *Topsentia genitrix*.^6^ The structures of these natural products were elucidated *via* spectroscopic methods, as shown in Fig. 1, and 1a–c were identified as weakly cytotoxic for fish and for dissociated cells of the freshwater sponge *Ephydatia fluviatilis*.^5^ After these initial discoveries, the structurally related analogues 1d–h and 2a–e, were isolated from various marine sponges, including *Spongosorites*,^2,5,7,8^*Hexadella*,^9,10^*Discodermia calyx*,^11^*Rhaphisia lacezie*,^12^ and *Topsentia*.^5,13^ Natural products of the Topsentin and Spongotine classes have been shown to possess cytotoxic, anticancer, antibacterial, antiviral, antifungal and anti-inflammatory activities.^3,14^

In addition to these previously discussed natural products, the most recently isolated natural product in the Spongotine class was Dihydrospongotine C (3), which was isolated as a single enantiomer in 2017 from the *Topsentia* sp. marine sponge. Its structure was elucidated *via* spectroscopic methods. Both stereocenters were determined to have the *S*-stereochemistry configurations consistent with experimental and calculated circular dichroism (CD) data (MPW1PW91/6-31G(d,p)).^13^ In addition, 3 displayed antibacterial activity toward *S. aureus* (MIC: 3.7 μg mL^−1^), anti-HIV activity (IC_50_ (YU2): 3.5 μM; IC_50_ (HxB2): 4.5 μM), and displayed no evidence of cytotoxic activity toward mammalian cells.^13^ Dihydrospongotine C (3) has yet to be accessed *via* total synthesis.

**Scheme 1:**
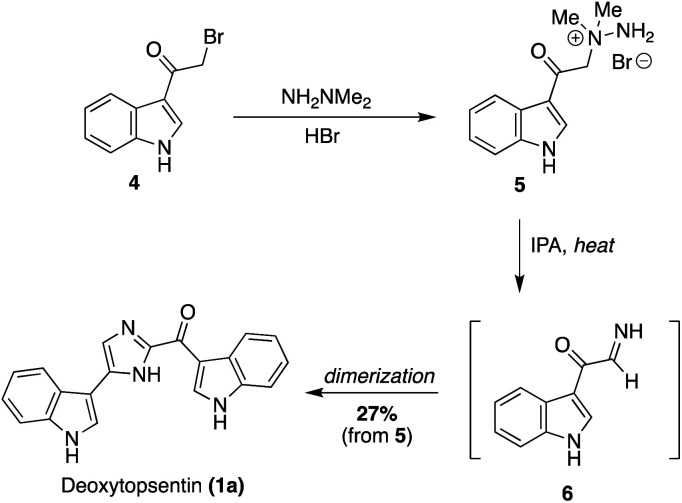
The first total synthesis of Deoxytopsentin (1a).

The first natural product of the Topsentin and Spongotine classes to be accessed *via* total synthesis was Deoxytopsentin (Topsentin A) (1a) by Braekman, J., *et. al.* in 1988.^15^ As shown in Scheme 1, 3-(bromoacetyl)indole (4) was reacted with 1,1-dimethylhydrazine in acidic conditions to afford the amine salt (5). Then, 5 was refluxed in isopropanol (IPA) to prompt migration of the methyl groups and elimination of dimethylamine to afford the imine intermediate 6, which immediately dimerized to afford 1a in 27% yield from 5.^15^

**Scheme 2:**
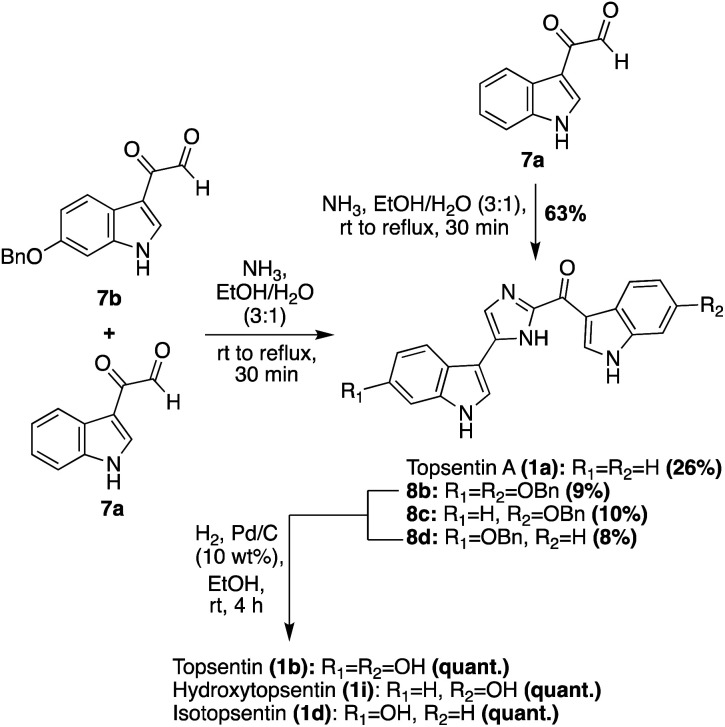
Total syntheses of Topsentins 1a–d, and the natural product analogue 1i.

Additional progress was then made toward the synthesis of the Topsentin natural products 1a–d, and the natural product analogue 1i. The synthetic route began with the synthesis of key indole-3-keto–aldehyde fragments 7a and 7b*via* a subsequent oxidative step that was carried out on the indole-3-α-chloro-ketone starting material. The penultimate step of this synthesis was the condensation and cyclization of 7a and 7b to ideally afford Topsentin (1b), as shown in Scheme 2. However, this cyclization proved to be unselective, affording a mixture of 1a (26%), 8b (9%), 8c (10%), and 8d (8%). Each of these intermediates were isolated before undergoing quantitative hydrogenolysis to remove the benzyl group, affording 1b, 1d, and the natural product analogue 1i. The same condensation/cyclization reaction was carried out to dimerize and condense two equivalents of 7a, rather than the mixed keto–aldehyde intermediates, and the desired product 1a was accessed in 63% yield. This emphasized that the selectivity was the major issue with this approach.^16^ Over the years, additional dimerization-cyclization approaches toward the total synthesis of 1a have also been completed, such as the dimerization of an indolic α-amino-ketone fragment by Miyake *et. al*.^17^

**Scheme 3:**
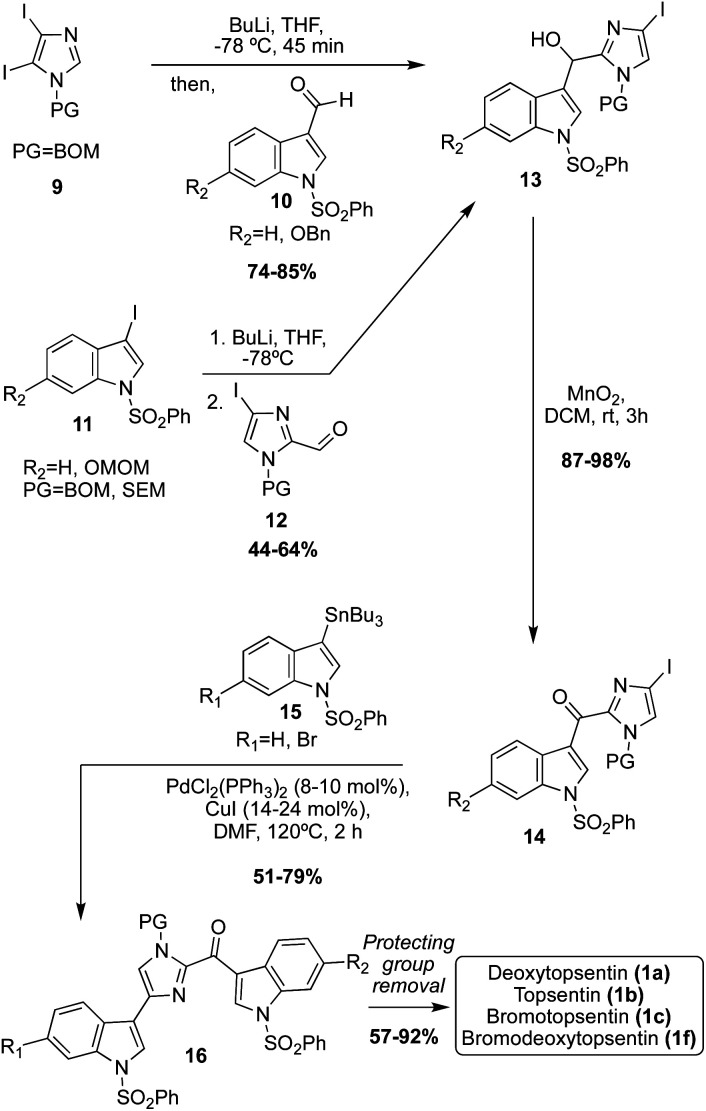
Lithiation and cross-coupling synthetic approach toward 1a–c, and 1f.

Considering the selectivity issues of these dimerization-like approaches toward the Topsentin natural products, additional synthetic approaches toward these scaffolds were developed to improve this selectivity, such as the use of lithiation and subsequent cross coupling reactions. For example, in this approach, the imidazole core was the starting point, and the indole-3-aldehyde and indole moieties were subsequently added to the imidazole ring (Scheme 3).^18,19^ There were two lithiation and subsequent nucleophilic addition approaches to access the key imidazoline intermediate (13). In the first synthetic strategy, di-iodo-imidazole (9) was first lithiated with *n*BuLi, which subsequently underwent nucleophilic addition to indole-3-aldehydes (10) to access 11 in high yields. In the second approach, the electronics of the reaction were reversed in which the 3-iodo-indoles (11) underwent lithium–halogen exchange and were subsequently added to the imidazole-2-aldehydes (12) to afford the key intermediate 13 in good yield. To access the Topsentin natural products 1a–c, and 1f, the alcohol 13 was oxidized to the ketone 14 using MnO_2_ and coupled with the tributyltin indole 15*via* the Stille method in high yield. After removal of protecting groups, the desired products 1a–c, and 1f were isolated in 57–92% yield.^18,19^ This approach proved to be a much more efficient and highly selective approach toward installing two indole fragments on the imidazole core that bear different substituents. Considering the efficiency of cross-coupling, additional cross-coupling approaches toward these natural products were completed.

**Scheme 4:**
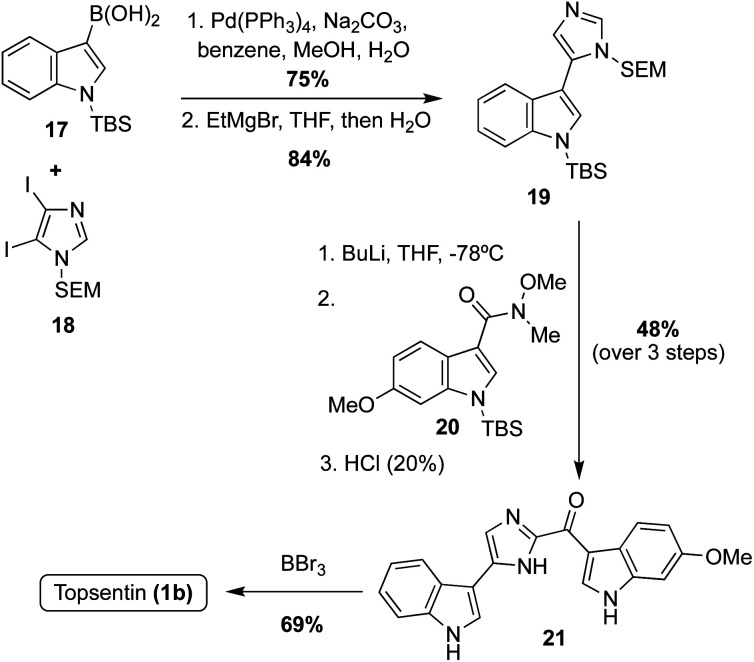
Cross-coupling and late-stage lithiation approach to Topsentin (1b).

For example, Kawasaki *et al.* coupled a borylated indole 17 with *N*-SEM-di-iodoimidazole 18 and subsequently de-iodinated to afford the imidazole intermediate 19 in high yields (Scheme 4). A late-stage lithiation of 19 was then carried out and subsequently reacted with the indole-3-amide intermediate 20. Lastly, the protecting groups were removed using BBr_3_, to render Topsentin (1b) in 69% yield.^20^

**Scheme 5:**
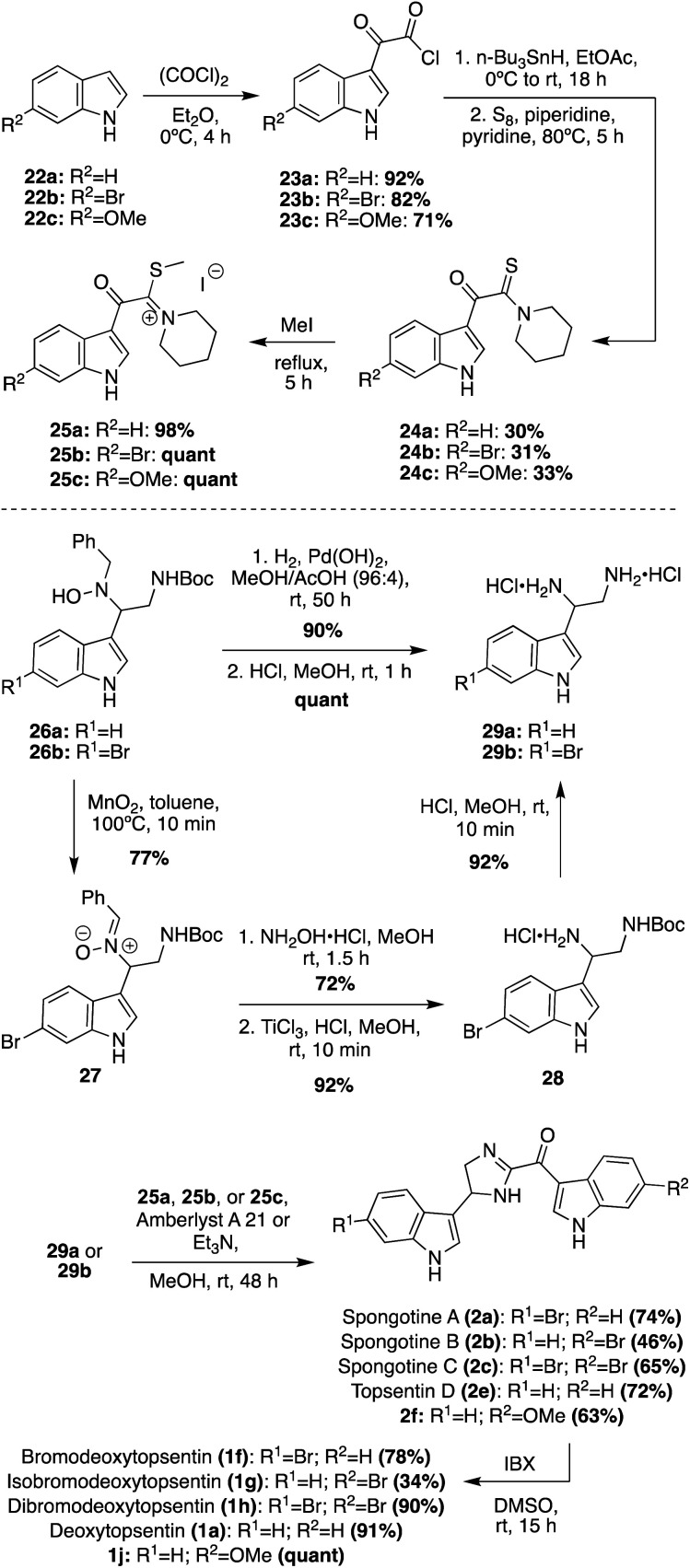
Total syntheses of 1a, 1f–h, 2a–c and 2e.

The newer isolated Spongotine natural products were accessed *via* total synthesis more recently. The first total syntheses of several Spongotine and Topsentin natural products were achieved *via* a common key cyclization approach toward the imidazoline core, in which a keto–thioimidate (25a–c) fragment and diamine (29a–b) fragment were cyclized.^21,22^ Intermediates 25a–c were synthesized in four steps from commercially available indole starting materials (22a–c) in good yields (Scheme 5). There were two different approaches for accessing the indolic diamine fragments (29a–b). The first approach was used to synthesize the indolic diamine (29a). In this approach, an indolic hydroxylamine intermediate (26a) underwent reductions and hydrogenolysis *via* hydrogen and Pearlman's catalyst to access the debenzylated intermediate in 90% yield. Then, the Boc protecting group was quantitatively removed under acidic conditions to access 29a.^23^ The second approach was a little more step-intensive to access the indolic diamine intermediate 29b as it could not be synthesized *via* the hydrogenative method of 29a due to problematic dehydrohalogenation of indoles under hydrogenative conditions.^23^ In order to avoid hydrogenation conditions, the 6-bromo-indolic hydroxylamine intermediate 26b was oxidized to 27*via* MnO_2_ and subsequently reacted with hydroxylamine hydrochloride to remove the benzyl group and reduced *via* TiCl_3_ to achieve 28 in high yields. From there, 28 was subjected to acidic conditions to remove the Boc group and access 29b in 92% yield.^22^ The desired natural products were then accessed *via* the key base-catalyzed imidazoline cyclization between 25a–c and 29a–b. This cyclization was carried out with either Et_3_N or Amberlyst A21 resin to access the desired natural products (2a (74%), 2b (46%),2c (65%), 2e (72%)) and the natural product analogue (2f (63%)) in good yields. Then, the imidazoline cores of 2a–c, 2e, and 2f were oxidized *via* IBX to the imidazole of Topsentins 1f (78%), 1g (34%), 1h (90%), 1a (91%), and Topsentin analogue 1j (quant). This was the first total synthesis of Spongotine A–C (2a–c), Bromodeoxytopsentin (1f), Isobromodeoxytopsentin (1g), and Dibromodeoxytopsentin (1h).^21,22^ The natural product analogue 1j was also synthesized and could be de-methylated in the future with BBr_3_ to access Hydroxytopsentin (Scheme 5).

**Scheme 6:**
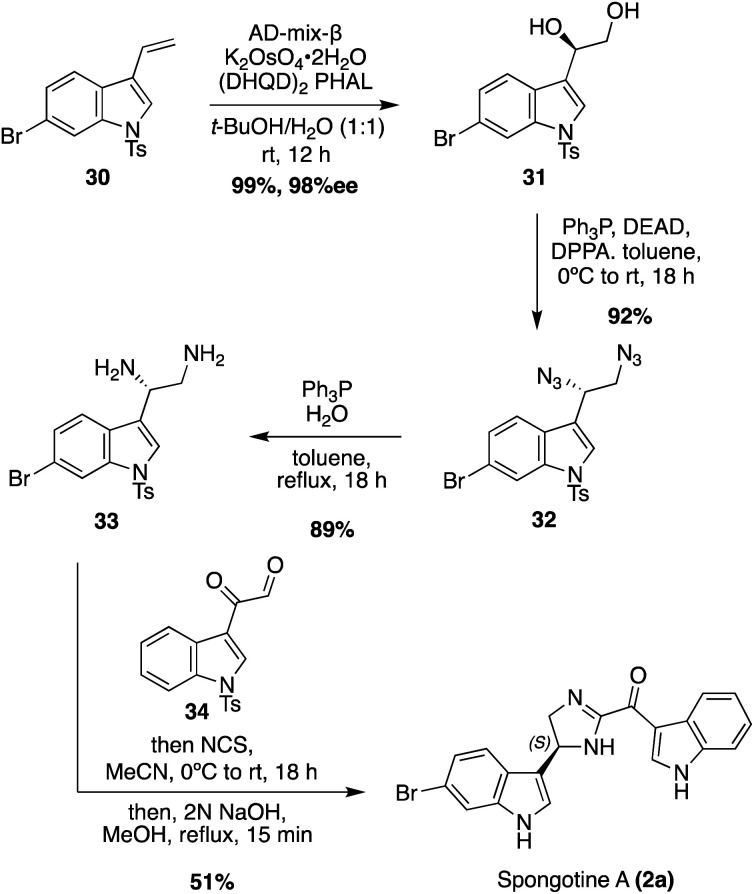
The First enantioselective total synthesis of (*S*)-Spongotine A (2a).

The first enantioselective total synthesis of Spongotine A (2a) was achieved *via* a key imidazoline cyclization between a keto–aldehyde fragment (34) and an optically active diamine fragment (33), as shown in Scheme 6.^24^ First, 34 was synthesized in three steps from 1*H*-indole, *via* a keto–acyl chloride intermediate, according to literature procedures.^25^ Fragment 33 was synthesized *via* Sharpless dihydroxylation of a 3-vinyl indole intermediate 30 to access (*R*)-31 in high yield and enantioselectivity. Then, (*R*)-31 underwent a stereospecific Mitsunobu reaction to access the diazide intermediate (*S*)-32 in 95% yield, in which the (*S*)-stereochemistry was set *via* inversion of the chiral center. The diazide (*S*)-32 was then reduced to access the optically active indolic diamine intermediate (*S*)-33 in high yields with 98% ee. To synthesize the imidazoline core and achieve Spongotine A (2a), (*S*)-33 and 34 underwent condensation, cyclization, and oxidation to the imidazoline *via* NCS. Subsequent removal of the tosyl protecting group with base achieved Spongotine A (2a) in 51% yield. Through these final steps, the stereochemistry was retained, allowing for the first enantioselective total synthesis of Spongotine A (2a).^24^ In addition, the specific optical rotation of the synthesized (*S*)-Spongotine A (2a) matched that of the natural Spongotine A (2a), allowing for the establishment of its previously unknown absolute configuration as (*S*)-Spongotine A.

#### 2.1.2. Nortopsentins

**Fig. 2:**
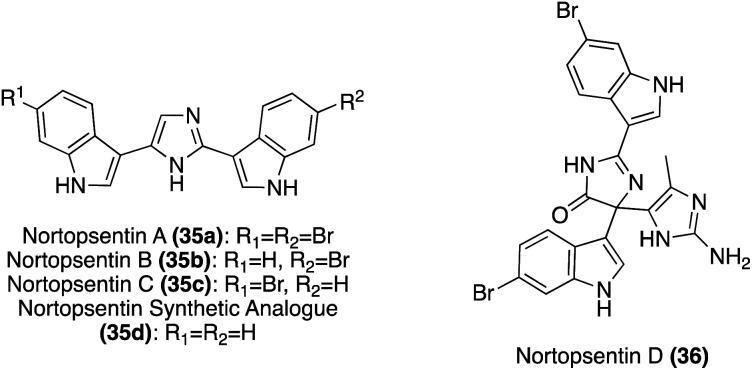
Chemical structures of Nortopsentins A–D (35a–c, 36) and Nortopsentin synthetic analogue (35d).

The Nortopsentin class of bis-indole natural products are structurally similar to those of the Topsentin class, however, they lack a carbonyl moiety on the imidazole linker moiety, as shown in Fig. 2. Nortopsentin A (35a), B (35b), and C (35c) were first isolated from *Spongosorites ruetzleri* in 1987.^26^ Nortopsentins A–C (35a–c) were found to possess cytotoxic and antifungal activities. Interestingly, the methylated derivatives of 35a–c also displayed enhanced cytotoxic activity in P388 cells compared to the isolated natural products.^26^ In addition, the unnatural synthetic Nortopsentin analogue 35d, unfortunately also referred to as Nortopsentin D in several early literature reports, was accessed *via* hydrogenation of 35a–c, as indole readily undergo de-halogenation under hydrogenation conditions.^27^ Several years later, in 1996, the more complex structural variant of this class, Nortopsentin D (36), composed of a tri-substituted imidazolinone ((4*H*)-imidazol-4-one) core, was first isolated from the axinellid sponge, *Dragmacidon* sp.^28^ Later, 36 was also isolated from the sponge *Agelas dendromorpha*.^29^ It is interesting to mention that the methylated derivative of 36 was also shown to have antifungal activity against yeast and high cytotoxicity toward tumoral cells.^28,30^

**Scheme 7:**
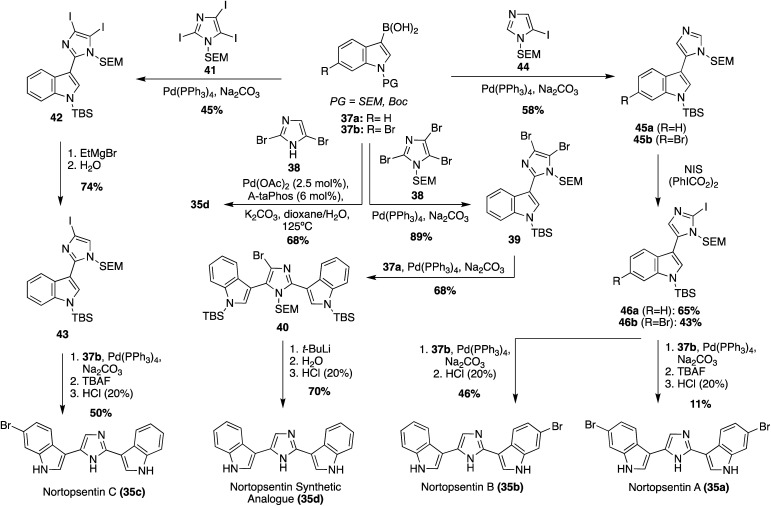
The first total syntheses of Nortopsentin A–C (35a–c), and the Nortopsentin synthetic analogue (35d).

The first total syntheses of Nortopsentins A–C (35a–c), as well as the synthetic analogue 35d, were achieved *via* a successive Pd-catalyzed cross-couplings of indole fragments to the imidazole core, as shown in Scheme 7.^30^ The synthetic analogue 35d was the first of these to be accessed *via* successive cross coupling reactions of a tri-brominated imidazole (38) with an *N*-TBS-protected indole-3-boronic acid (37a), using Pd(PPh_3_)_4_ as a catalyst. The protecting groups and remaining bromide were then removed to access 35d in high yields. During route development, SEM- and MOM-protecting groups were also explored for 37a, yet the TBS-protecting group resulted in the highest yield of the coupling reaction.^30^ More recently, an adapted method for palladium-catalyzed cross coupling of unprotected imidazoles was developed and utilized in the synthesis of 35d, as shown in Scheme 7.^31^ This Suzuki–Miyaura cross-coupling method allowed for a significantly expedited approach toward 35d, as compared to previous reports. However, this method is likely only efficient for natural products bearing symmetrical indole moieties. In a similar manner, the first total syntheses of Nortopsentin A (35a) and C (35c) were achieved, as shown in Scheme 7. The efficient synthesis of 35c was very similar to that of 35d. Iodide was utilized as a coupling partner here, rather than bromide. Iodide was likely used here to prevent potential selectivity issues given the bromide substituents on the boronic acid coupling partners. The other major difference was the removal of the additional iodide before the second indole coupling (Scheme 7, synthesis of 35c). It is noteworthy to mention that the selective de-iodination to access intermediate 43 was confirmed *via* a NOESY correlation between the SEM group on the nitrogen and the C5–H of the imidazole.^30^ The approach toward the first total synthesis of Nortopsentin A (35a) and Nortopsentin B (35b), which also bear bromide substituents on their indoles, implemented a later stage iodination approach in the synthesis of 46 before the second cross coupling reaction to access 35a and 35b in moderate and good yield, respectively (Scheme 7).^30^

**Scheme 8:**
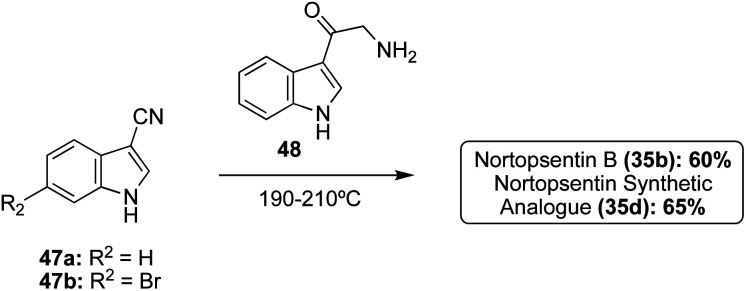
Syntheses of Nortopsentin B (35b) and its synthetic analogue (35d) *via* thermal condensation–cyclization.

In addition to these cross-coupling approaches, a different non-cross-coupling approach was used to access the Nortopsentin natural products 35b and 35d. This method utilized the condensation of a nitrile with an α-amino-ketone fragment and subsequent cyclization and aromatization of the imidazole at high temperatures to achieve Nortopsentin B (35b) and the Nortopsentin D synthetic analogue 35d in good yields (Scheme 8).^17^ This method was not viable for the total synthesis of Nortopsentins A (35a) and C (35c). This was likely due to the necessary hydrogenation step for the synthesis of intermediate 48, which would not tolerate a bromide substituent on the indole (48), as has been previously discussed.

**Scheme 9:**
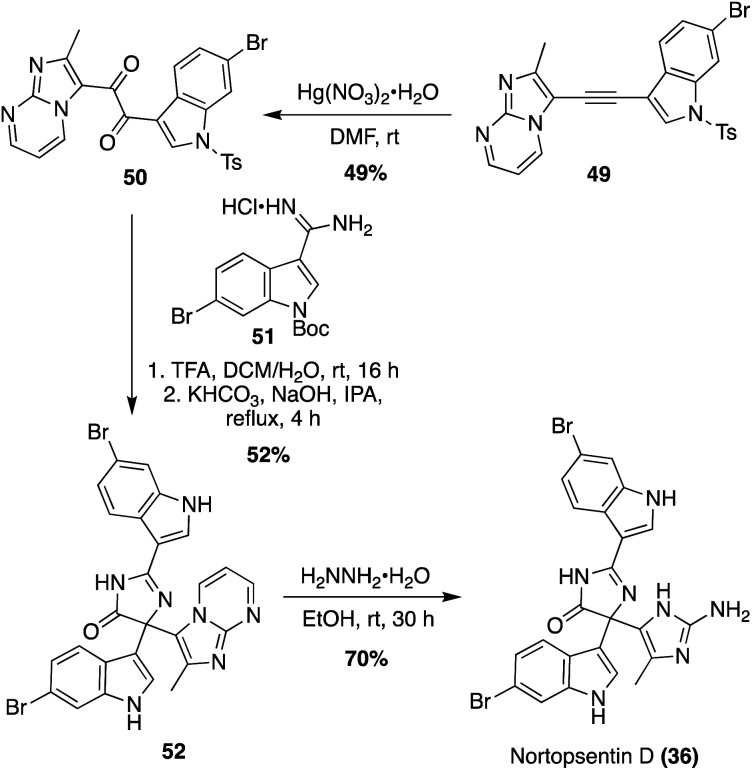
First total synthesis of Nortopsentin D (36).

The first total synthesis of the more structurally complex Nortopsentin D (36) was completed by K. Keel, *et. al.*, in 2021, as shown in Scheme 9.^32^ The complex tri-substituted imidazolinone ((4*H*)-imidazol-4-one) core was constructed *via* a late-stage Pinacol-like rearrangement. Two key fragments were utilized in this approach; an alkyne fragment (49) and amidine fragment (51). The alkyne (49) and amidine (51) were accessed in two and five steps, respectively from the commercially available 6-bromo-1*H*-indole.^32^ The alkyne fragment (49) was oxidized to the di-ketone intermediate (50) *via* mercuric nitrate monohydrate. The key condensation–cyclization and subsequent Pinacol-like rearrangement steps were then carried out to form the tri-substituted imidazolinone core of Nortopsentin D (36), accessing 52 in 52% yield. The *N*-tosyl and *N*-boc protecting groups were deprotected during this reaction. Precise deprotection was key for optimization, as successful cyclization was dependent upon electronics. It was found that the *N*-boc protecting group of 51 had to first be deprotected to allow for sufficient nucleophilicity of the amidine (51) to condense and cyclize with the di-ketone (50). The presence of the *N*-tosyl protecting group of 50 was also found to contribute to increased electrophilicity of the ketone. Therefore, initial acidic conditions were implemented to remove the *N*-boc group of 51, followed by the strategic use of a mildly nucleophilic base and solvent to allow for cyclization to occur prior to de-tosylation of 50 (Scheme 9). After removal of the remaining protecting group to access the 2-amino-imidazole substituent, Nortopsentin D (36) was accessed in 70% yield.^32^

### 2.2 Imidazolinone linker moieties

#### 2.2.1. Rhopaladins

**Fig. 3:**
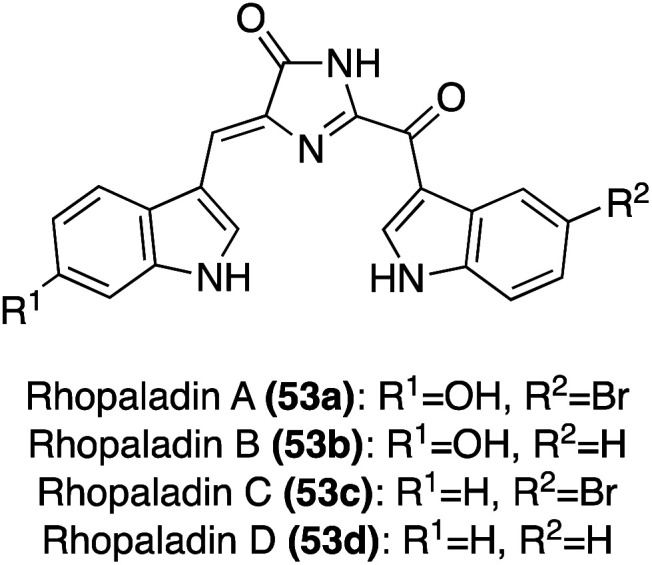
Chemical structure of Rhopaladins A–D (53a–d).

Natural products of the Rhopaladin class are bis-indole alkaloids comprised of two indole fragments connected by an imidazolinone ((4*H*)-imidazol-4-one) linker moiety, similar to Nortopsentin D (36). In addition, the indolylcarbonyl substitution at the C-2 position of the imidazolinone core is similar to the Spongotine and Topsentin natural product classes (Fig. 3). However, unlike any of the previously discussed natural product classes, the second indole moiety is connected at the 5-position of the imidazolinone core by a unique vinyl chain. Rhopaladins A–D (53a–d) were first isolated in 1998 by Sato *et. al.* from the marine tunicate *Rhopalaea* sp.^33^ The geometry of the alkene present in these natural products (53a–d) was identified as (*Z*) by a NOESY experiment that was run on Rhopaladin C (53d). The studies identified various NOESY correlations, such as a NOESY correlation between the two C-2 hydrogens of the indole moieties, indicating a (*Z*) geometry of the alkene. In terms of biological activities, these natural products were reported to demonstrate antibacterial activity against *Sarcina lutea* and *Corynebacterium xerosis* and inhibitory activity against cyclin dependent kinase 4 and c-erbB-2 kinase. However, their broader biological activity has yet to be explored.^33^

**Scheme 10:**
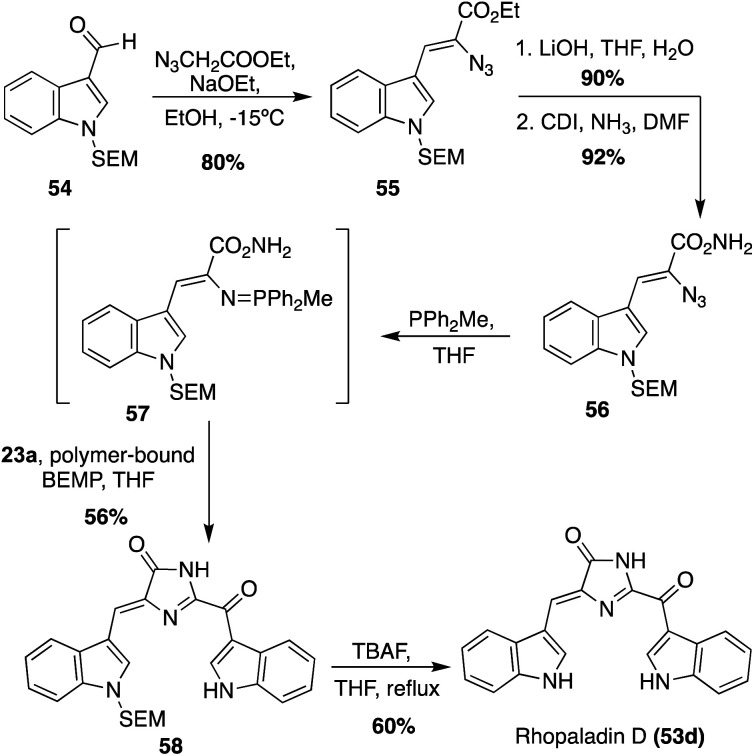
First total synthesis of Rhopaladin D (53d).

The first total synthesis of Rhopaladin D (53d) was reported in 2000, in which a key intermolecular aza-Wittig reaction was utilized, followed by subsequent condensation and cyclization to form the imidazolinone core of 53d.^34^ To perform the aza-Wittig reaction, the azide intermediate (56) was first accessed in three steps in high yields from indolyl-3-aldehyde 54 (Scheme 10). Then, the aza-Wittig reaction was performed on azide intermediate 56 to access intermediate 57, which was immediately condensed and cyclized with keto–acyl chloride 23a to access imidazolinone 58 in 56% yield over two steps. Lastly, the protecting group was removed to afford Rhopaladin D (53d) in 60% yield. However, 58 was isolated as a 6 : 4 mixture of *E*/*Z* isomers and after chromatographic separation, the (*Z*) isomer isomerized to the (*E*) isomer upon sunlight irradiation. Therefore, after the last step, Rhopaladin D (53d) was also isolated as a mixture of *E*/*Z* isomers.^34^

**Scheme 11:**
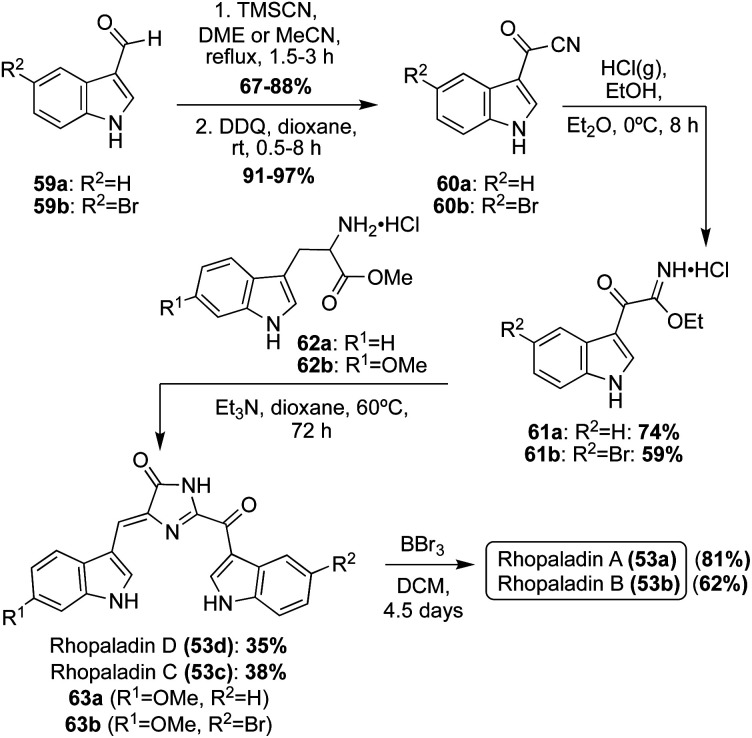
Total syntheses of Rhopaladins A–D (53a–d).

A couple years later, the total syntheses of all four Rhopaladins A–D (53a–d) were achieved *via* a key imidate-based cyclization with tryptophan esters to form the imidazolinone core of these natural products, as shown in Scheme 11.^35^ First, the carbonyl nitrile intermediate (60a–b) was synthesized *via* a TMS-cyanohydrin intermediate from aldehyde 59a–b, followed by subsequent oxidation to the carbonyl nitrile 60a–b in high yields. Then, 60a–b underwent a Pinner reaction with gaseous hydrochloric acid and ethanol to form the imidate intermediate (61a–b). Compounds 61a–b were immediately condensed and cyclized with tryptophan methyl ester hydrochloride (62a) in the presence of triethylamine to access Rhopaladin C (53b) and D (53d) in 38% and 35% yield, respectively. Interestingly, the dehydrogenation to form the alkene of the Rhopaladins occurs spontaneously after the cyclization has occurred. This is likely due to the highly conjugated nature of the Rhopaladins, contributing to high stability. Interestingly, this cyclization and spontaneous dehydrogenation specifically produced (*Z*)-isomers of the Rhopaladins, as was confirmed *via* NOESY correlations, including a NOESY correlation between the C2–H's of the indole moieties. The presence of the (*E*)-isomers could not be detected.^35^ Thus, this method was proven to be very selective to the (*Z*)-isomer, which is a major advantage compared to the previous synthesis of Rhopaladin D (53d). Rhopaladins A and B (53a and 53b) were synthesized in the same manner as Rhopaladin C and D (53c and 53d), as shown in Scheme 11. The only difference was the identity of the tryptophan methyl ester (62b). In addition, after the key cyclization reaction and spontaneous dehydrogenation to access 63a and 63b, an additional de-methylation step *via* BBr_3_ was required to access Rhopaladins A (53a) and B (53b) in 81% and 62% yields, respectively. In addition, similarly to 53c and 53d, the Rhopaladins A (53a) and B (53b) were confirmed with NOESY as the desired (*Z*)-isomer.^35^

#### 2.2.2. Spongosoritins

**Fig. 4:**
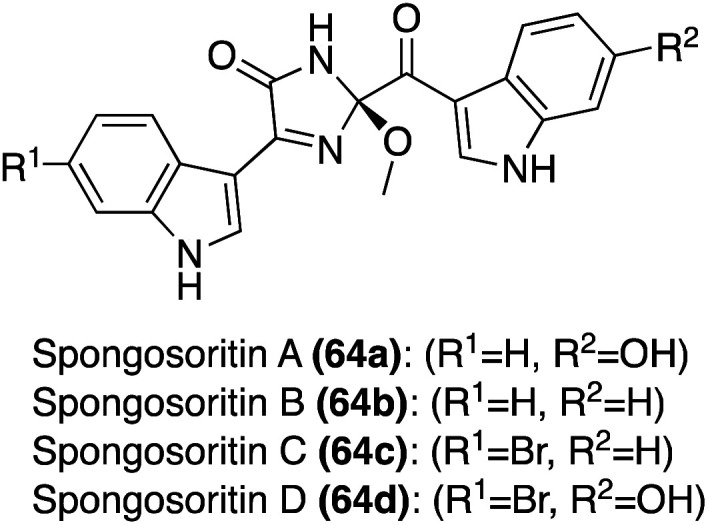
Chemical structures of Spongosoritins A–D (64a–d).

Recently, the first of the Spongosoritins were isolated, which marks another natural product class to contain an imidazolinone (2,3-dihydro-4*H*-imidazol-4-one) core. Spongosoritins A–D (64a–d) were first isolated in 2021 from *Spongosorites* sp. by Park, *et. al*.^36^ As shown in Fig. 4, the chemical structure of the Spongosoritins contains one indole moiety and one indolyl-3-ketone moiety connected by a 2-methoxy-1-imidazole-5-one linker. These chemical structures were elucidated *via* spectroscopic methods. In addition, these Spongosoritins (64a–d) contain one stereocenter at the C-2 position of the imidazolinone. The absolute configuration of this position was determined *via* a density functional theory (DFT)-based computational method and electronic circular dichroism (ECD). Comparing measured data with the calculated data, the stereochemistry was assigned as a (2*R*) configuration.^36^

Initial biological activity exploration of the Spongosoritins (64a–d) was conducted and it was found that they exhibited moderate inhibition against transpeptidase sortase A and weak inhibition against human pathogenic bacteria and A549 and K562 cancer cell lines. The biological activity findings are detailed in Table 1, including the known antibiotic, ampicillin, for comparison.^36^ Though some information is now known, much of the Spongosoritins' biological activities remain elusive. Furthermore, Spongosoritins A–D (64a–d) have yet to be accessed *via* total synthesis.

**Table 1:** Biological activity of Spongosoritins A–D (64a–d)

	Gram (+) MIC (μg mL^−1^)			Gram (−) MIC (μg mL^−1^)		IC_50_ (μM)			
#	*S. aureus*	*Enterococcus faecalis*	*Enterococcus faecium*	*Klebsiella pneumonia*	*Salmonella enterica*	*E. coli*	Srt A	A549	K562
64a	>128	>128	>128	>128	>128	>128	>329.8	77.3	24.2
64b	64	>128	>128	128	128	>128	62.7	55.7	28.2
64c	32	128	>128	>128	64	>128	43.9	61.2	37.7
64d	16	120	120	>128	64	>128	>274.7	70.9	54.2
Ampicillin	0.13	0.5	1	—	0.25	8			

#### 2.2.3. Violaceins

**Fig. 5:**
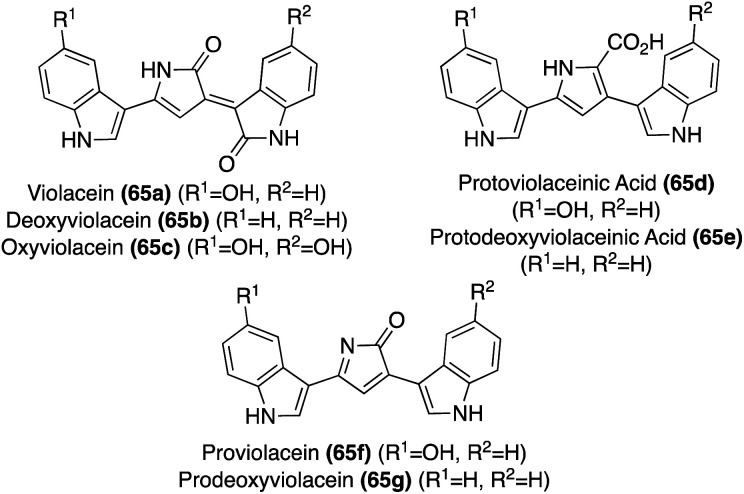
Chemical structures Violacein (65a) and related natural product analogues (65b–g).

Natural products of the Violacein class are purple-blue pigments produced from bacteria, unlike many other bis-indole natural products which come from marine sponges and tunicates. Violacein (65a) was first discovered in 1882 by Boisbaudran, *et. al*.^37^ Natural products of the Violacein class (65a–g) have been isolated from several Gram-negative bacteria, including *Chromobacterium violaceum*, *Janthionobacterium lividum*, *Pseudoalteromonas luteoviolacea*, *Psudomonas* sp., *Collimonas* sp., *Dunganella*. etc.^38–42^ As shown in Fig. 5, the structure of the Violacein natural products consist of three main sub-classes, the Violaceins (65a–c), whose two indole moieties are connected by a 1,3-dihydro-2*H*-pyrrol-2-one core linker, the Protoviolaceinic acids (65d–e), whose two indole moieties are connected by a pyrrole-2-carboxylic acid linker, and the Proviolaceins (65f–g), which contain a 2*H*-pyrrol-2-one core. These various sub-classes are produced from the same biosynthetic pathway, either as related paths or as intermediates toward one another.^43^ In addition, these natural products exhibit significant biological activity, such as antibacterial, antifungal, anticancer, antiviral, and antiparasitic activity.^44^

**Scheme 12:**
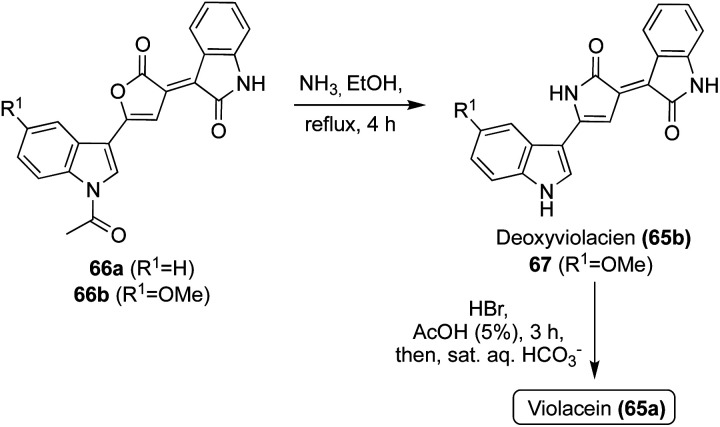
Total syntheses of Violacein (65a) and Deoxyviolacein (65b).

Natural products of the Violacein class (65a–g) have been most widely accessed *via* exploration of biosynthetic pathways.^43^ However, these biosynthetic pathways fall outside the scope of this review. Progress has also been made toward the total synthesis of these compounds. The first total synthesis of Violacein and Deoxyviolacein was achieved by Ballantine, *et. al.*^45^*via* a key reaction of the lactone intermediates 66a or 66b with ammonia under heating to replace the oxygen atom of the lactone with a nitrogen atom to afford the desired 1,3-dihydro-2*H*-pyrrol-2-one core of Deoxyviolacein (65b) and imidazolinone intermediate (67) in good yields, as is shown in Scheme 12. Violacein was then accessed after an additional de-methylation step of 67 to complete the first total synthesis of Violacein (65a).^45^

**Scheme 13:**
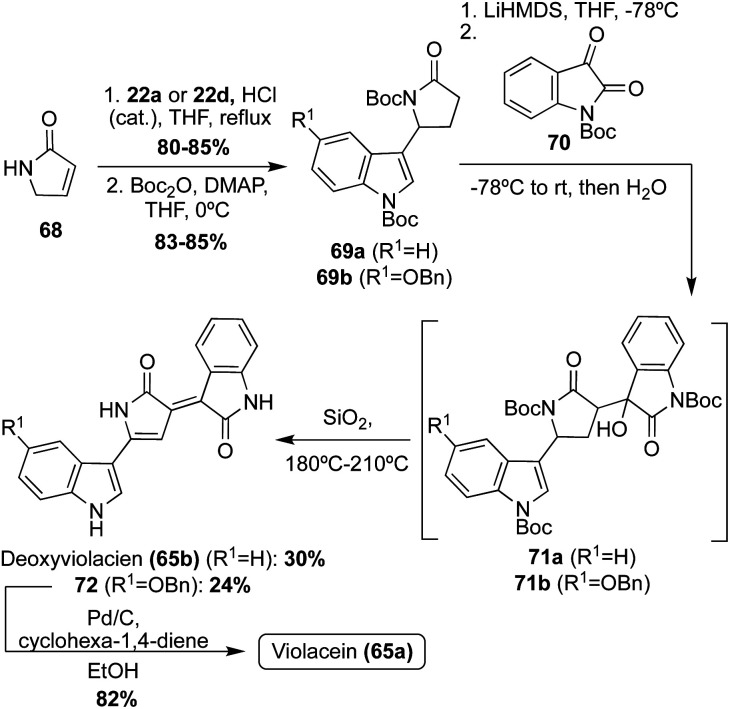
Total syntheses of Violacein (65a) and Deoxyviolacein (65b).

Violacein and Deoxyviolacein were also accessed *via* total synthesis in 2001, in which Steglich, *et. al.* implemented a strategy where the two indole groups were attached stepwise to an already formed pyrrolinone core, as shown in Scheme 13.^46^ First, pyrrolinone 68 underwent an acid-catalyzed reaction with 22a or 22d, and subsequent protection of both the nitrogens *via* Boc-anhydride, to access intermediates 69a–b in high yields. Then, 69a–b were converted to their corresponding enolates using LiHMDS as the base, followed by subsequent reaction with *N*-Boc-isatin (70) to afford the aldol condensation intermediates (71a–b). Then, 71a–b were immediately heated to afford Deoxyviolacein (65b) and intermediate 72 in 30% and 24%, respectively. It is noteworthy to mention that upon adsorption to silica gel and heating for 15–20 minutes, dehydration, dehydrogenation, and cleavage of the Boc-protecting groups all occurred simultaneously. Lastly, 72 underwent debenzylation to access Violacein (65a) in 82% yield. The configurations of 65a–b were confirmed as (*E*)-isomers *via* NMR.^48^ This approach was advantageous as it was significantly higher yielding than the first reported total synthesis of these natural products (65a–b).

**Scheme 14:**
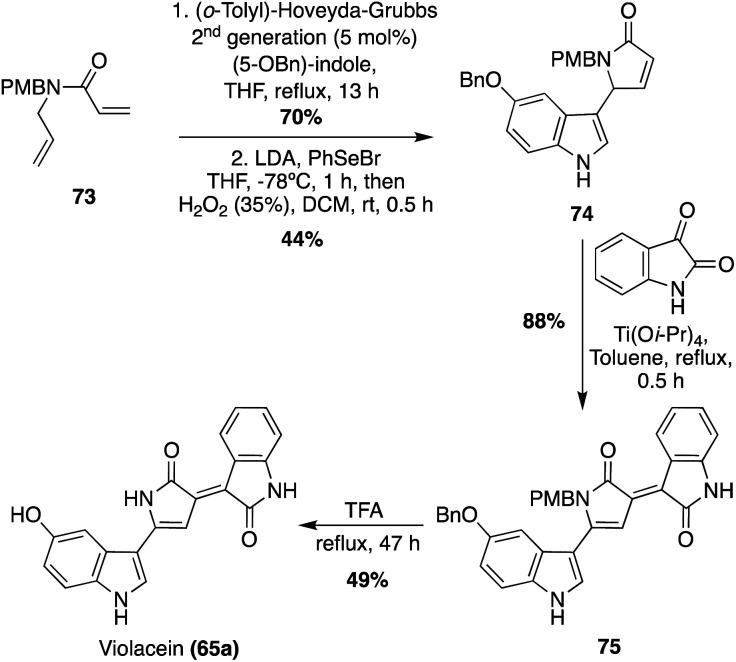
Total synthesis of Violacein (65a) *Via* a tandem RCM/isomerization/nucleophilic addition sequence.

A more recent approach used a tandem ring-closing metathesis (RCM)/isomerization/nucleophilic addition sequence toward the total synthesis of Violacein, as shown in Scheme 14.^47^ This tandem RCM/isomerization/nucleophilic addition was the first step used in the total synthesis, followed by elimination to intermediate 74 in good yields. The oxindole moiety was then attached *via* a Ti-catalyzed tautomerization/aldol condensation with isatin to access 75 in high yield. After acid-mediated removal of protecting groups, Violacein (65a) was achieved in 49% yield. The authors mentioned that the final product 65a was very difficult to handle and had very minimal solubility in common solvents. Therefore, it was suspected that the crude yield was significantly higher than the isolated yield.^47^

### 2.3 Pyrrole and pyrrole-dione linker moieties

#### 2.3.1. Arcyriarubins

**Fig. 6:**
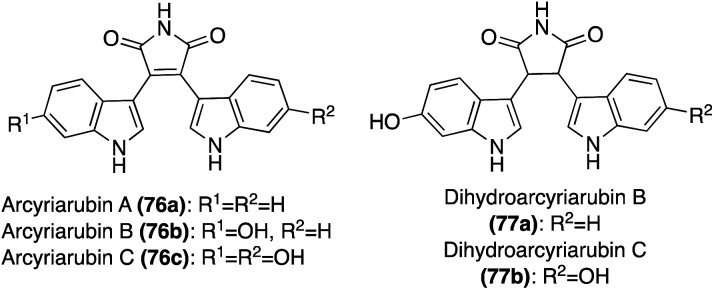
Chemical structures of Arcyriarubins A–C (76a–c) and Dihydroarcyriarubins A–B (77a–b).

Arcyriarubins A–C (76a–c) are bisindolylmaleimides first isolated by Steglich, *et. al.* in 1980 from the red sporangia of the slime mold *Arcyria denudate*.^48^ Arcyriarubins B (76b) and C (76c) were also isolated from *Tubifera casparyi* in 2003 by Ishibashi, *et. al.*^49^ The chemical structure (Fig. 6) of these compounds was elucidated by spectroscopic means. Arcyriarubins A–C (76a–c) contain two indole moieties linked by a pyrrole-2,5-dione (maleimide) core. These natural products display moderate antibiotic, antifungal, cytotoxic, and kinase inhibition activities.^48,49^ These natural products are also of great interest, as many structurally related bisindolylmaleimides are selective protein kinase C (PKC) inhibitors, which show promise as novel potential therapeutics for autoimmune diseases.^50,51^

In addition, two structurally related natural products that have also been isolated include Dihydroarcyriarubins B (77a) and C (77b). As shown in Fig. 6, these natural products are incredibly similar to the Arcyriarubins (76a–c), however, the reduced pyrrolidine-2,5-dione (succinimide) core of the Dihydroarcyriarubins (77a–b) sets them apart. Dihydroarcyriarubin B (77a) was first isolated by Steglich, *et. al.* from the slime mold, *Arcyria denudate*, alongside the Arcyriarubins (76a–b).^48^ Dihydroarcyriarubin C (77b) was first isolated in 2003 by Ishibashi, *et. al.* from *Arcyria ferruginae*, alongside Arcyriarubin C (76b).^49^ The biological activities of the Dihydroarcyriarubins B (77a) and C (77b) are very similar to that of 76a–c, including antibiotic, antifungal, cytotoxic, and kinase inhibition activities.^48,49,52^

**Scheme 15:**
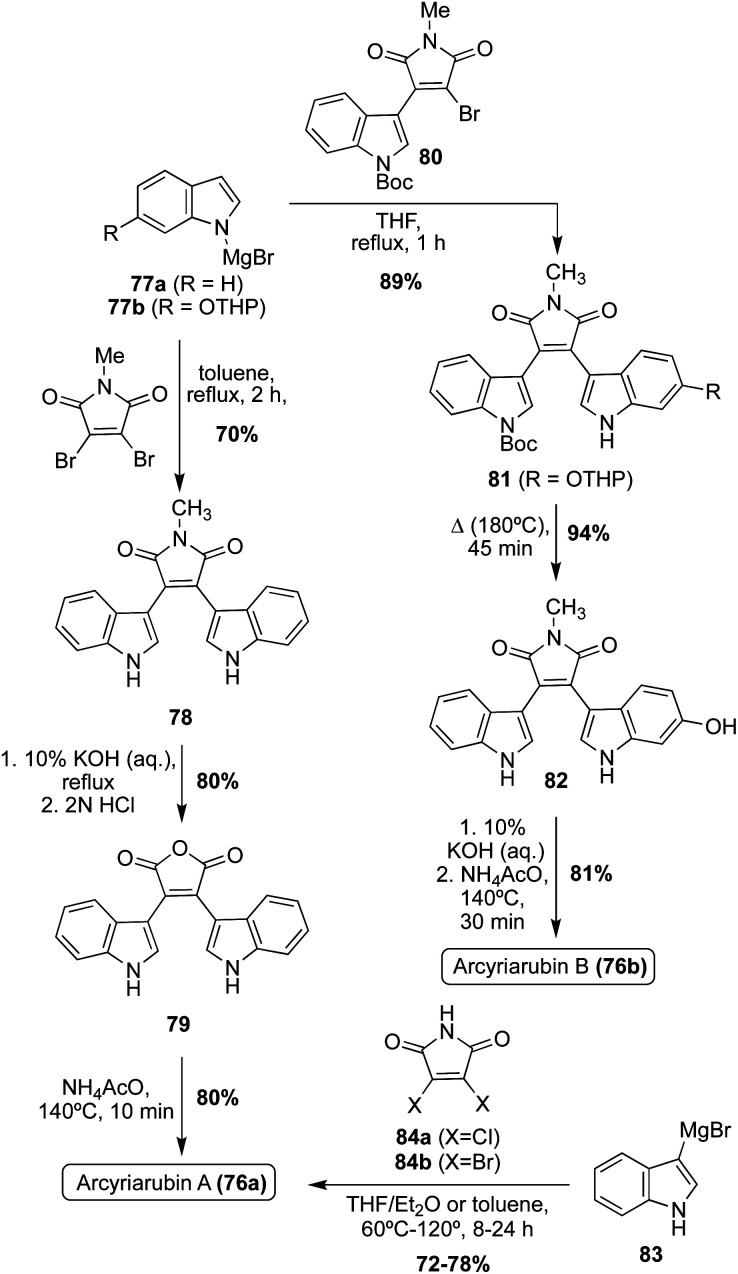
Total synthesis of Arcyriarubins A–B (76a–b).

The first natural products of the Arcyriarubin class to be accessed *via* total synthesis were Arcyriarubin A (76a) and Arcyriarubin B (76b) in 1988 by Steglich, *et. al*.^53^ These total syntheses were accomplished using Grignard reactions to install the indole moieties on the maleimide core, as shown in Scheme 15. In the first total synthesis of Arcyriarubin A (76a), a Grignard reaction was carried out *via* an indole Grignard reagent and 3,4-dibromo-*N*-methylmaleimide to access bisindolylmaleimide intermediate (78) in high yield. To remove the methyl group of 78, a maleic anhydride intermediate (79) was accessed and then subsequently reacted with ammonium acetate at high temperatures to access Arcyriarubin A (76a) in high yields.^53^ Due to the asymmetric nature of the indole moieties in Arcyriarubin B (76b), the synthetic approach to this natural product was slightly different. As shown in Scheme 15, the first total synthesis of 76b was achieved *via* a reaction of an indole Grignard reagent (77b) with a mono-indolic maleimide intermediate (80) to access intermediate 81 in 89% yield. This approach allowed for the synthesis of a maleimide intermediate with asymmetric indole moieties (81) in a stepwise manner. After de-methylation and removal of protecting groups, Arcyriarubin B (76b) was accessed in high yield.^53^

Several years later, a similar Grignard approach to the total synthesis of Arcyriarubin A (76a) was utilized in 1995 (Scheme 15).^54^ Here, an indole Grignard (83) was reacted with 3,4-dichloromaleimide (84a) at high temperature to access Arcyriarubin A (76a) in 72% yield. This approach was advantageous in that it allowed for the synthesis of Arcyriarubin A (76a) without the need for protecting groups, which greatly expediting its synthesis.^54^ However, the intolerance for asymmetric indole moieties in this method makes it unsuitable for application to other Arcyriarubins. This Grignard strategy was again slightly improved upon in 2017 for the total synthesis of Arcyriarubin A (76a), as shown in Scheme 15.^55^ Utilizing the same indole Grignard (83) and 3,4-dibromomaleimide (84b) at a higher temperature, for a shorter reaction time, Arcyriarubin A (76a) was synthesized in 78% yield.^55^ It is also noteworthy to mention that Arcyriarubin C (76c) has yet to be accessed *via* total synthesis.

**Scheme 16:**
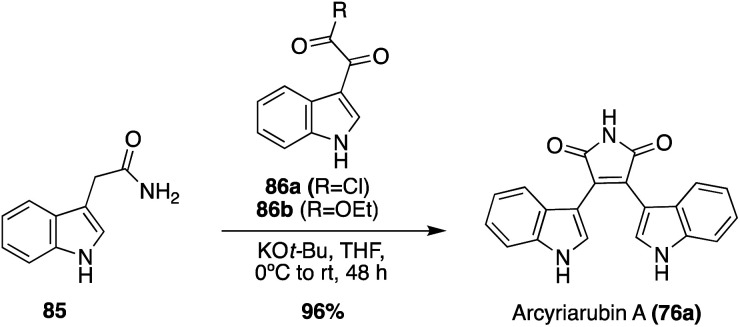
Total synthesis of Arcyriarubin A (76a).

In addition to this Grignard approach, a more biomimetic approach has also been explored toward the total synthesis of Arcyriarubin A (76a), as shown in Scheme 16. This approach consisted of a base-mediated condensation-cyclization reaction between an indole-3-acetamide intermediate (85) and an indole-3-oxo-acyl chloride intermediate (86a) to access Arcyriarubin A (76a), although in a low 11% yield. Recently, an adapted and improved biomimetic total synthesis of Arcyriarubin A (76a) was developed, as shown in Scheme 16. For this approach, intermediate 85 was condensed and cyclized with an indole-3-oxo-ester intermediate (86b) to access Arcyriarubin A (76a) in an excellent 96% yield.^56^

**Scheme 17:**
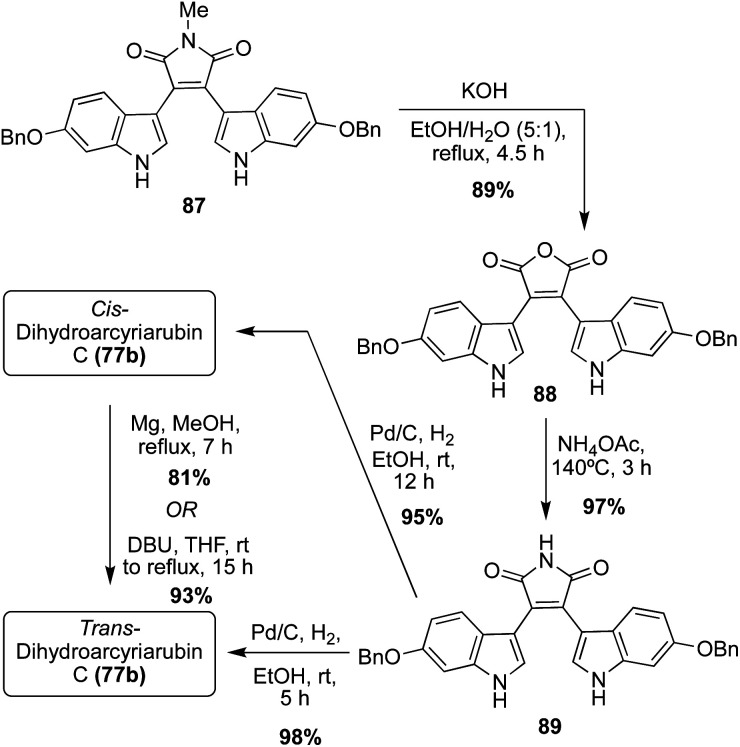
Total synthesis of Dihydroarcyriarubin C (77b).

In terms of the Dihydroarcyriarubins (77a–b), Dihydroarcyriarubin B (77a) has yet to be accessed *via* total synthesis. On the other hand, the first total synthesis of Dihydroarcyriarubin C (77b) was completed in 2007 by Ishibashi, *et. al.*, as shown below in Scheme 17.^52^ First, the bis-indolyl-*N*-methylmaleimide intermediate (87) was synthesized in four steps from 3,4-dibromomaleimide and 6-benzyloxyindole, following the same Grignard approach as was previously published by Steglich, *et. al.*^53^ To remove the *N*-methyl group, intermediate 87 was converted to the maleic anhydride intermediate (88), which was subsequently reacted with ammonium acetate at high temperatures to access the bis-indolylmaleimide intermediate (89) in very high yields. Then, 89 underwent hydrogenation to reduce the maleimide core of 89 to the succinimide core of Dihydroarcyriarubin C (77b). In addition to the desired reduction of 89, both benzyl groups were also removed. While this was beneficial for the total synthesis of Dihydroarcyriarubin C (77b); it was also the reason why this approach was not viable for synthesizing Arcyriarubin C (76c). It is noteworthy to mention that at a 12 hours hydrogenation reaction time, *cis*- Dihydroarcyriarubin C (77b) was accessed in 95% yield, but at a 5 hours reaction time, *trans*- Dihydroarcyriarubin C (77b) was accessed in 98% yield. In addition, *cis*- Dihydroarcyriarubin C (77b) could be isomerized to *trans*-Dihydroarcyriarubin C (77b) either by magnesium in methanol or DBU in tetrahydrofuran in 81% and 93% yield, respectively. To accomplish the first total synthesis of Dihydroarcyriarubin C (77b), both the *cis*-(77b) and *trans*-(77b) isomers were synthesized to allow for the stereochemical determination of the isolated Dihydroarcyriarubin C (77b). Comparison of their NMR data allowed for confirmation of the isolated 77b as *trans*-Dihydroarcyriarubin C (77b).^52^

#### 2.3.2. Lycogarubins

**Fig. 7:**
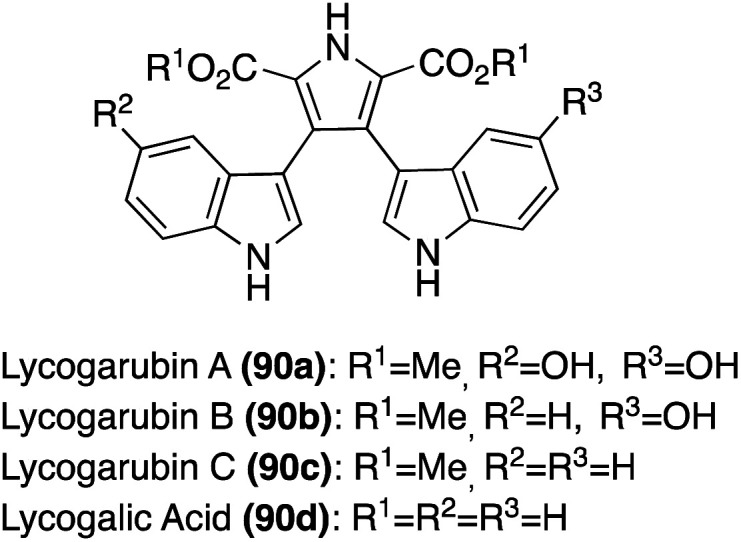
Chemical structures of Lycogarubins A–C (90a–c) and Lycagolic acid (90d).

Lycogarubins A–C (90a–c) and Lycagolic acid (90d) were first isolated independently by Steglich, *et. al.* and Asakawa, *et. al.* from *Lycogala epidendrum*, a slime mold (Myxomycetes).^57,58^ Lycagolic Acid (90d) has also been referred to as chromopyrrolic acid (CPA). The chemical structures of these natural products were elucidated *via* spectroscopic analysis. As shown below in Fig. 7, the two indole moieties of these bis-indole natural products are linked by a pyrrole-2,5-methyl ester core in Lycograubins A–C (90a–c) or a pyrrole-2,5-carboxylic acid core in Lycagolic acid (90d). These natural products closely resemble the previously discussed Arcyriarubin natural products (76a–c), differing mainly in the aromatic pyrrole core of 90a–d rather than the maleimide core of 76a–c. Much of the biological activity of these natural products is unexplored, though Lycogarubin C displayed moderate anti-HSV-I virus activity.^58^ In addition, these compounds are of interest due to their structural similarity to potent kinase inhibitors.^52^

**Scheme 18:**
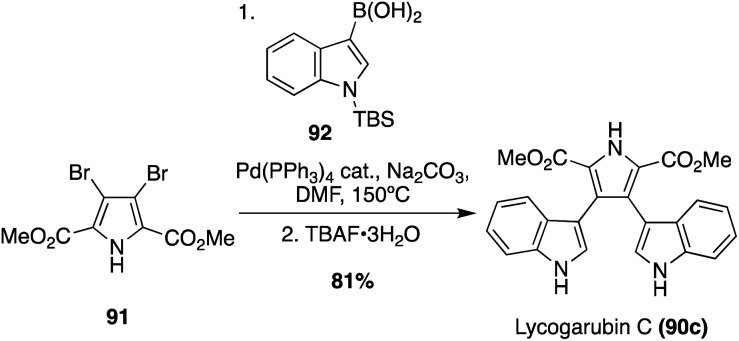
Total synthesis of Lycogarubin C (90c).

**Scheme 19:**
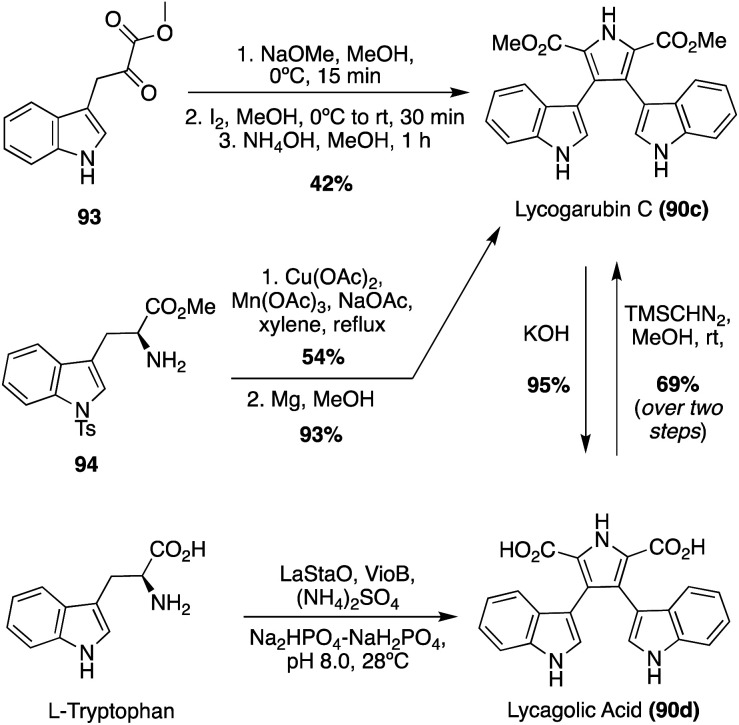
Biomimetic total syntheses of Lycogarubin C (90c) and Lycagolic acid (90d).

Many of the early syntheses of the Lycogarubins (90a–c) and Lycagolic acid (90d) were carried out *via* the exploration of the biosynthetic pathway to these natural products.^59^ However, the first of these natural products to be accessed *via* total synthesis was Lycogarubin C (90c) using a palladium-catalyzed Suzuki coupling reaction of dimethyl 3,4-dibromo-1*H*-pyrrole-2,5-dicarboxylate (91) with an indole-3-boronic acid (92) and subsequent removal of the protecting group *via* TBAF to access Lycogarubin C (90c) in 81% yield, as shown in Scheme 18.^35^ This Suzuki method has been implemented in recent literature as well in the synthesis of 90c and similar structures.^60^ Considering the ongoing study of the biosynthesis of the Lycogarubin natural products, several biomimetic syntheses have been developed as well. One of the earliest of these was completed in 2006 in which a methyl 3-(1*H*-indol-3-yl)-2- oxopropanoate intermediate (93) underwent a dimerization/cyclization sequence over three steps to access Lycogarubin C (90c) in 42% yield (Scheme 19).^59,61^

Another biomimetic synthesis of Lycogarubin C (90c), as well as Lycagolic acid (90d), was carried out *via* a Cu/Mn co-oxidized dimerization/cyclization of a Tosyl-tryptophan methyl ester (94) with another molecule of itself (Scheme 19). It is noteworthy to mention that biosynthesis of the Lycogarubins also begins with tryptophan starting materials. After removal of the protecting groups, Lycogarubin C (90c) was accessed in high yields. Lycogarubin C (90c) was then converted to Lycagolic acid (90d) in 95% yield *via* base-catalyzed ester hydrolysis.^62^

Recently, a semi-total synthesis of Lycogarubin C (90c) was completed *via* Lycagolic acid (90d) as an intermediate (Scheme 19). First, enzymatic biological reactions were conducted to accomplish the dimerization/cyclization of the l-Tryptophan (l-Trp) starting material to access Lycagolic acid (90d). Then, 90d was immediately reacted with TMSCHN_2_ in methanol to access Lycogarubin C (90c) in 69% over two steps from the simple and readily accessible l-Trp starting material.^63^

**Scheme 20:**
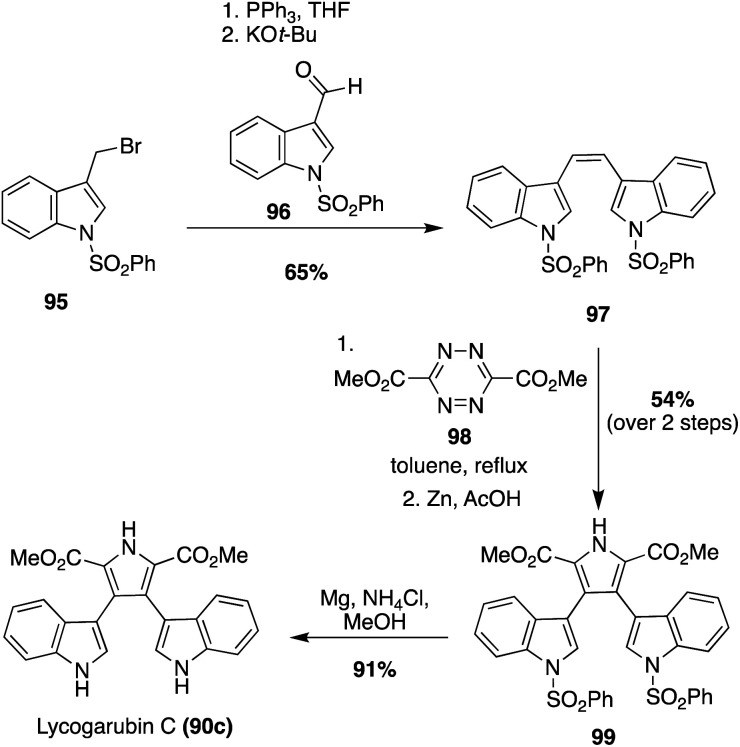
Total synthesis of Lycogarubin C (90c).

Another synthetic method that has been used to synthesize Lycogarubin C (90c) and Lycagolic acid (90d) was the use of key Diels–Alder and Kornfeld–Boger ring contraction reactions. One example utilizing this method, as shown in Scheme 20, began with the synthesis of the di-protected-bis-indole alkene intermediate (97) *via* a Wittig reaction between the bromide intermediate (95) and the aldehyde intermediate (96). Then, 97 underwent a Diels–Alder reaction with the tetrazine intermediate (98), followed by a subsequent Kornfeld–Boger ring contraction to access the pyrrole intermediate (99) in 54% yield over two steps. After removal of the protecting groups, Lycogarubin C (90c) was accessed in 91% yield. Protection of both indole moieties was necessary to access intermediate 99 in good yields and to avoid side product formation *via* the Diels–Alder/Kornfeld–Boger reaction sequence.^64^

**Scheme 21:**
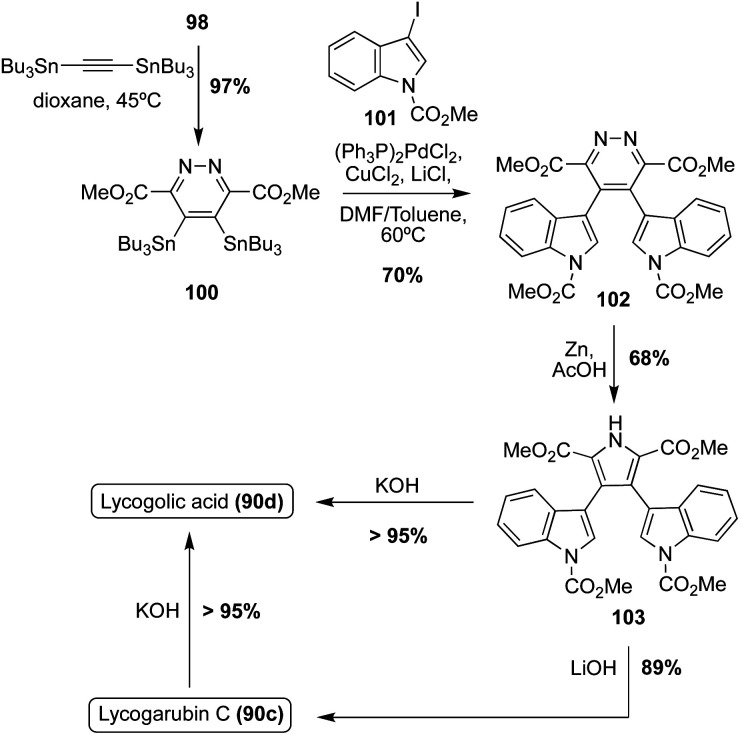
Total syntheses of Lycogarubin C (90c) and Lycagolic acid (90d).

Another example in which this method was implemented, as shown in Scheme 21, began with the Diels–Alder reaction between 98 and 1,2-bis-(tributylstannyl)-acetylene to access diazine intermediate (100) in very high yield. Stille coupling of 100 with the 3-iodo-indole intermediate (101) and a subsequent Kornfeld–Boger ring contraction of the diazine core led to the desired pyrrole core of 103 in good yields. Then, 103 could be subjected to LiOH or KOH to access Lycogarubin C (90c) or Lycagolic acid (90d), respectively, in very high yields. Furthermore, Lycogarubin C (90c) could also be converted to Lycogolic acid (90d) *via* KOH in very high yields.^65^ This approach (Scheme 21) is favorable in comparison to the previously discussed Diels–Alder/Kornfeld Boger approach (Scheme 20), as it not only allowed for the total synthesis of Lycogarubin C (90c) in good yields, but also allowed for the total synthesis of Lycagolic acid (90d) in good yields.

#### 2.3.3. Lynamicins

**Fig. 8:**
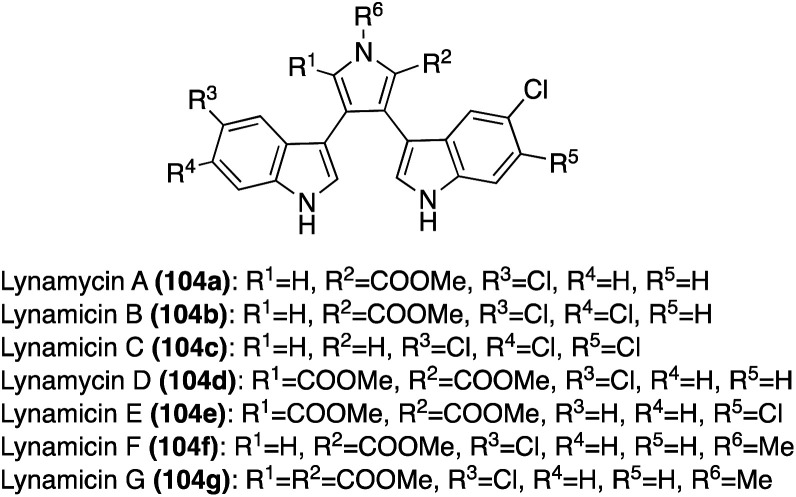
Chemical structures of Lynamicin A–G (104a–g).

More recently, a class of natural products that is structurally related to the Lycogarubins (90a–d) was discovered. In 2008, Lynamicns A–E (104a–e) were isolated by Potts, *et. al.* from a novel marine actinomycete, of the proposed genus *Marinispora*. The chlorinated bis-indole chemical structures linked by a pyrrole moiety, as shown in Fig. 8, was confirmed in these natural products *via* spectroscopic methods.^66^ Not only were these natural products (104a–e) the first examples of bis-indole pyrrole alkaloids bearing chloride substituents, but the Lynamicins A–E (104a–e) have also been found to display broad-spectrum antibiotic activity against both Gram-positive and Gram-negative bacteria, such as *Staphylococcus aureus* and *Enterococcus faecium*.^66^ The Lynamicins have also been shown to possess anticancer and kinase inhibition activity.^67^

Two additional natural products of the Lynamicin class, Lynamicin F (104f) and Lynamicin G (104g), were isolated in 2014 by Zhang, *et. al.*^68^ from the deep-sea-derived actinomycete, *Streptomyces* sp. The chemical structures of Lynamicin F (104f) and Lynamicin G (104g), as shown in Fig. 8, were elucidated *via* spectroscopic methods. The characteristic *N*-methyl group in 104f and 104g was confirmed *via* key HMBC correlations between the hydrogens on the methyl group and the C-2 and C-5 carbons of the pyrrole ring. Interestingly, the presence of this methyl group seems to have a significant effect on biological activity because, unlike Lyncamicins A–E (104a–e), Lynamicin F (104f) and Lynamicin G (104g) showed no significant antibiotic activity against *Staphylococcus aureus*, *Escherichia coli*, *etc.* and no significant cytotoxic activity toward cancer cell lines.^68^

**Scheme 22:**
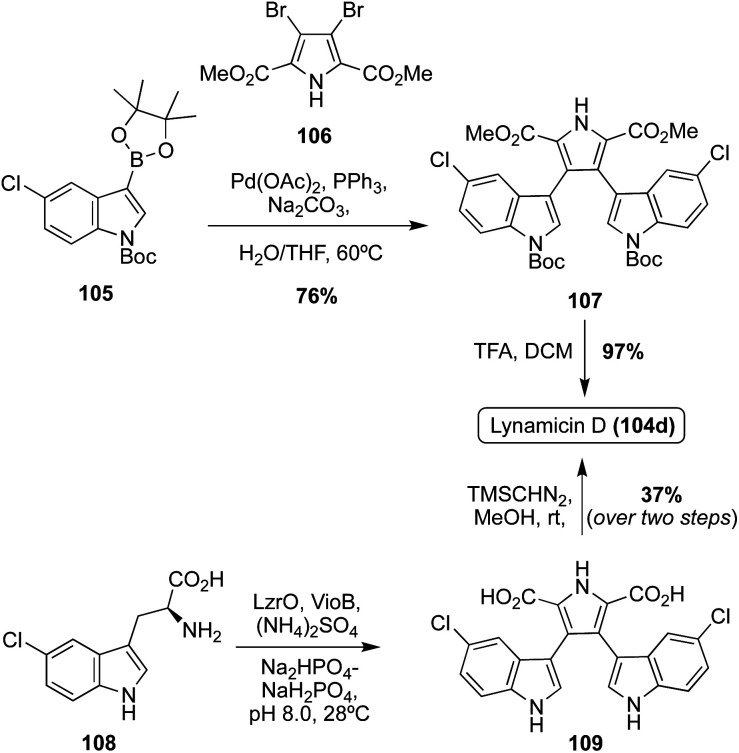
Total synthesis of Lynamicin D (104d).

The first of the Lycogarubin class to be accessed *via* total synthesis was Lynamicin D (104d) by Nikolakaki, *et. al*. in 2017, which utilized a Suzuki coupling approach to install the two indole moieties to the pyrrole core.^68^ As shown in Scheme 22, first, the 3,4-dibromopyrrole intermediate 106 was initially synthesized in two steps from the corresponding pyrrole-2-methylester in good yields. An indolic boronic ester intermediate (105) then underwent a palladium-catalyzed Suzuki reaction with 106 to access intermediate 107 in 76% yield. After removal of the Boc protecting groups *via* acidic conditions, Lynamicin D (104d) was achieved in 97% yield.^68^ Recently, a semi-total synthesis of Lynamicin D (104d), resembling that of Lycogarubin C (90c), was also completed (Scheme 22).^63^ First, enzymatic biological reactions were conducted to accomplish the dimerization/cyclization of the 5-chloro-l-Tryptophan (5-Cl-l-Trp) starting material (108), following the biosynthetic path, to access the carboxylic acid analogue of Lynamicin D (109). Then, 109 was immediately reacted with TMSCHN_2_ in MeOH to access Lynamicin D (104d) in 37% over two steps from the simple 5-Cl-l-Trp (108) starting material.^63^

**Scheme 23:**
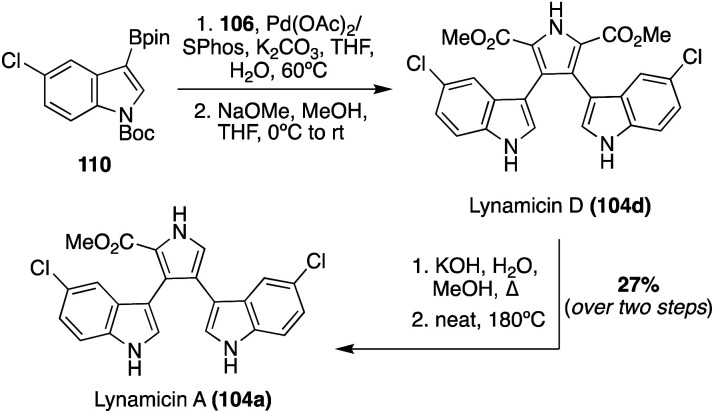
Total synthesis of Lynamicin A (104a).

The first total synthesis of Lynamicin A (104a) was completed in 2021 by Smith, *et. al.*, as well as a slightly adapted total synthesis of Lynamicin D (104d).^69^ As shown in Scheme 23, these total syntheses follow a very similar Suzuki cross-coupling to that of Nikolakaki's previously discussed method to install the indole moieties onto the pyrrole core of these natural products.^67^ However, the Suzuki cross coupling step between the boronic ester intermediate (110) and the 3,4-dibromo pyrrole intermediate (106) utilized different palladium catalysts, as shown in Scheme 23, and featured a subsequent deprotection of the protecting groups to access Lynamicin D (104d) in 67% yield. Then, Lynamicin D (104d) was subjected to a basic environment, at high temperatures, which led to the removal of one of the ester groups on the pyrrole ring, accessing Lynamicin A (104a) in 67% yield.^69^ Lynamicins B, C, and E–G (104b, c, e–g) have yet to be accessed *via* total synthesis.

### 2.4 Piperazine, piperazinone, and pyridone linker moieties

#### 2.4.1. Dragmacidins

**Fig. 9:**
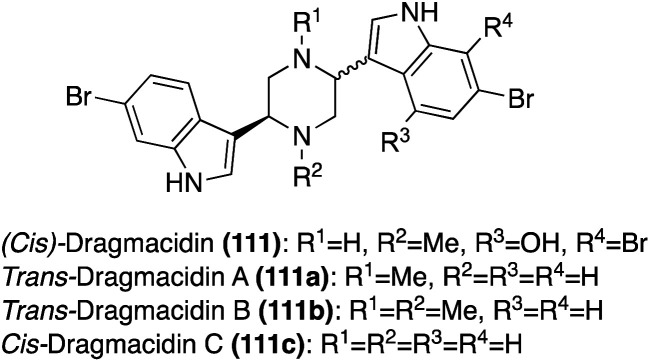
Chemical structures of Dragmacidin (111) and Dragmacidins A–C (111a–c).

The Dragmacidin class of natural products consists of two main structural sub-classes. The first sub-class consists of Dragmacidin (111) and Dragmacidins A–C (111a–c), which are bis-indole alkaloids whose two indole moieties are linked by a piperazine core, as shown in Fig. 9. Dragmacidin was first isolated in 1988, by Koehn *et. al.*, from *Dragmacidon* marine sponge.^70^ A couple years later, Dragmacidin A (111a) and Dragmacidin B (111b) were isolated from *Hexadella* marine sponge.^10^ The chemical structure of these natural products was elucidated *via* spectroscopic methods. Dragmacidin (111) and Dragmacidins A–B (111a–b) were confirmed to have *trans* configurations.^10,71^ Dragmacidin C (111c) was the last of this sub-class to be discovered and was first isolated in 1991, by Smith, *et. al.*, from the sea squirt *Didenium candidum*.^71^ Dragmacidin C (111c) was initially thought to have *trans* configuration, but it was later shown to be of *cis* configuration.^72^ The biological activities of Dragmacidin (111) and Dragmacidins A–C (111a–c) are relatively unexplored; however, Dragmacidin (111) and Dragmacidin A (111a) have been found to display significant cytotoxicity against a variety of cancer cell lines.^70^

**Fig. 10:**
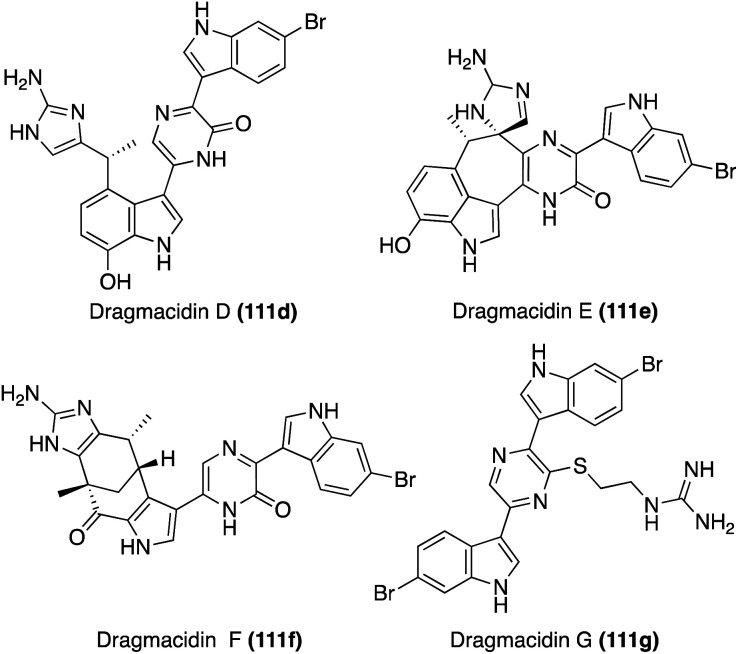
Chemical structures of Dragmacidins D–G (111d–g).

The second sub-class of the Dragmacidins is comprised of more structurally complex natural products, including Dragmacidins D (111d), E (111e), and F (111f). These bis-indole natural products (111d–f) contain a 2-pyridone core that links their two indole, or modified indole, moieties, as shown in Fig. 10. Another structural distinction of this sub-class is the presence of a guanidine moiety. Dragmacidin D (111d) and Dragmacidin E (111e) were isolated in 1988 by Carroll, *et. al.* from *Spongosorites* and *Hexadella* marine sponges and their structures were elucidated *via* spectroscopic means.^73^ In addition, Dragmacidin D (111d) and Dragmacidin E (111e) were found to be potent inhibitors of serine–threonine protein phosphatases.^73^ Dragmacidin F (111f) was isolated a couple years later, in 2000, by Riccio, *et. al.* from a *Halicortex* marine sponge. The complex ring structure of Dragmacidin F (111f) was elucidated *via* extensive spectroscopic analysis and its unprecedented carbon skeleton was proposed to result from the cyclization of a partially oxidized form of Dragmacidin D (111d). Dragmacidin F (111f) was found to display antiviral activity against HSV-1 and HIV-1.^74^ Dragmacidin E (111e) and Dragmacidin F (111f) have both been accessed *via* total synthesis; however, these syntheses will not be discussed in detail as these chemical structures fall outside the scope of this review.^75,76^

Recently, Dragmacidin G was isolated by Wright, *et. al.* from *Spongosorites* marine sponge. As is shown in Fig. 10, the chemical structure of Dragmacidin G (111g) does not exactly fit into the second sub-class of the Dragmacidins, as it is the only natural product in its class to contain a piperazine (1,4-diazine) core linking the two indole moieties. However, Dragmacidin G (111g) does contain a characteristic guanidine moiety, thus enabling It to still be considered part of the second structural sub-class of the Dragmacidins. In addition, Dragmacidin G (111g) was found to exhibit antibacterial activity against *Stapphylococcus aureus*, *Mycobacterium tuberculosis*, *Plasmodium falciparum*, *etc.*, and cytotoxic activity against a panel of pancreatic cell lines.^77^ Dragmacidin G has yet to be accessed *via* total synthesis.

**Scheme 24:**
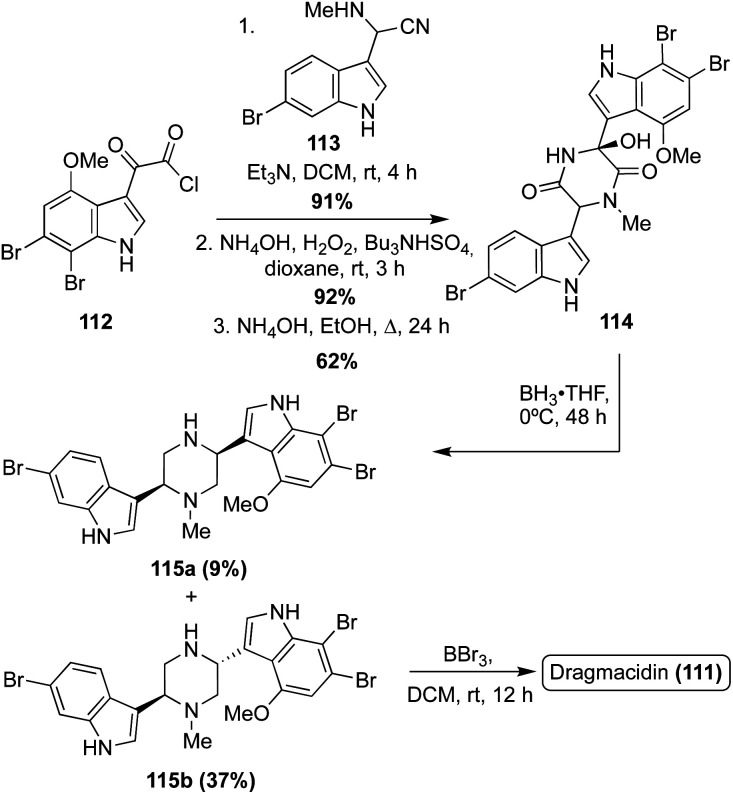
First total synthesis of Dragmacidin (111).

Dragmacidin (111) was the first natural product of this class to be accessed *via* total synthesis. In 1994, Wuonola *et. al.* completed the first racemic total synthesis of Dragmacidin (111) *via* an initial condensation of an oxo-acyl chloride (112) and an amino nitrile (113) (synthesized *via* a Strecker method), followed by subsequent oxidation and cyclization, as shown in Scheme 24. These transformations proceeded in high yields. The cyclized intermediate was then subjected to reductive conditions to access 115a and 115b. This reduction, specifically the loss of the hydroxyl group, was not stereoselective at room temperature, resulting in a ratio of (115a : 115b = 1 : 1). However, significant selectivity in favor of the desired *trans* isomer (115b) was achieved by lowering the temperature to 0 °C for this reduction reaction, as shown in Scheme 24, resulting in 37% of the *trans* isomer (115b) compared to 9% of the undesired *cis* isomer (115a). Then, 115b was de-methylated to afford Dragmacidin (111).^78^

**Scheme 25:**
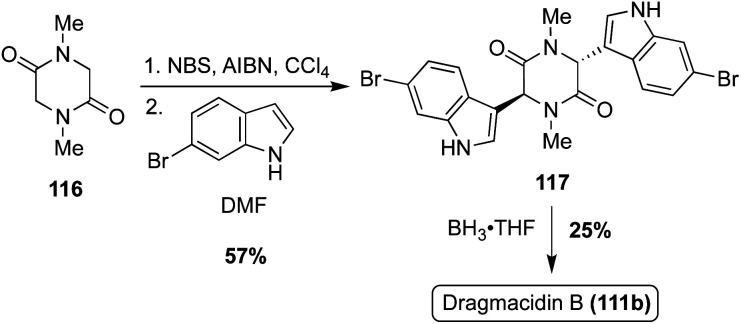
First total synthesis of Dragmacidin B (111b).

The first total synthesis of Dragmacidin B (111b) was achieved in the same year as Dragmacidin (111). However, considering its less substituted indole moieties, the approach toward Dragmacidin B (111b) was less step intensive, as shown in Scheme 25. First, an *N*-dimethylated piperazinedione (116) was brominated before undergoing a double nucleophilic attack with 6-bromo-1*H*-indole to access intermediate 117 in 57% yield. The bromination and nucleophilic attacks were conducted in a one-pot manner and no acid or base was necessary for the nucleophilic step. Then, 117 was reduced to access Dragmacidin B (111b) in moderate yield. This made for a very expeditious two-step approach to complete the total synthesis of Dragmacidin B (111b) in 14% yield overall (Scheme 25).^79^

**Scheme 26:**
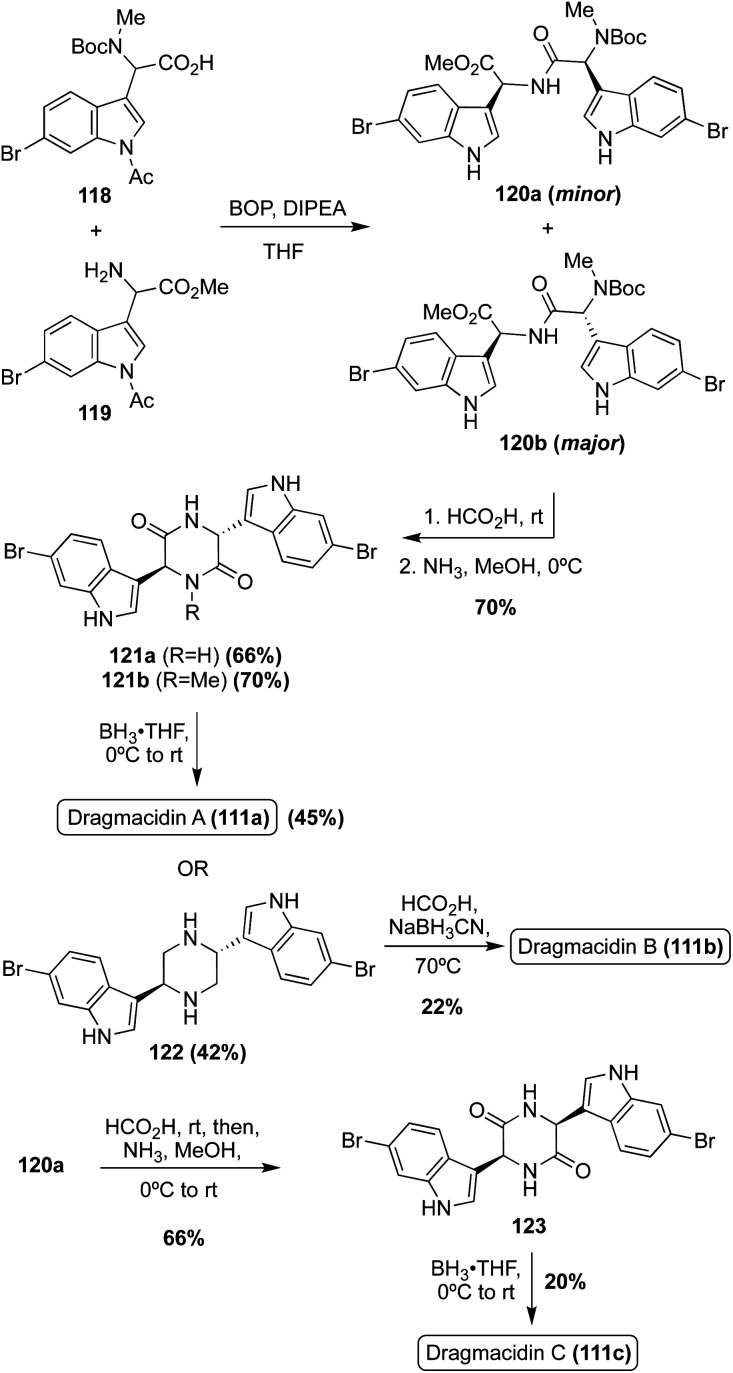
Total syntheses of Dragmacidins A–C (111a–c).

The first total synthesis of Dragmacidin A (111a) was completed in 2000 *via* a condensation/cyclization sequence of methylated (118) and free (119) indolic α-amino carboxylic acids to access the piperazinedione intermediate (121) in good yields, as shown in Scheme 26. The condensation step was diastereoselective and the desired isomer (120b) predominated. After the cyclization, the carbonyl groups of intermediate (121a) were removed *via* reductive conditions to access Dragmacidin A (111a) in 45% yield. The total yield of Dragmacidin A (111a) after this efficient three-step approach was 21% overall.^80^

The first total synthesis of Dragmacidin C (111c) was completed in 2002 *via* the same three step method (condensation/cyclization/oxidation) that was previously discussed for the first total synthesis of Dragmacidin A (111a), and the relative *cis* configuration of Dragmacidin C (111c) was confirmed (Scheme 26). As shown in Scheme 26, Dragmacidin B (111b) was also synthesized using this method, though incomplete reduction of 121b resulted in a prominent side product in which one carbonyl remained on the ring. This accounts for the lower yield of 122. In the synthesis of Dragmacidin B (111b) a late-stage methylation step was carried out on the piperazine intermediate (122) to install the two methyl groups on the piperazine ring of Dragmacidin B (111b).^72^

**Scheme 27:**
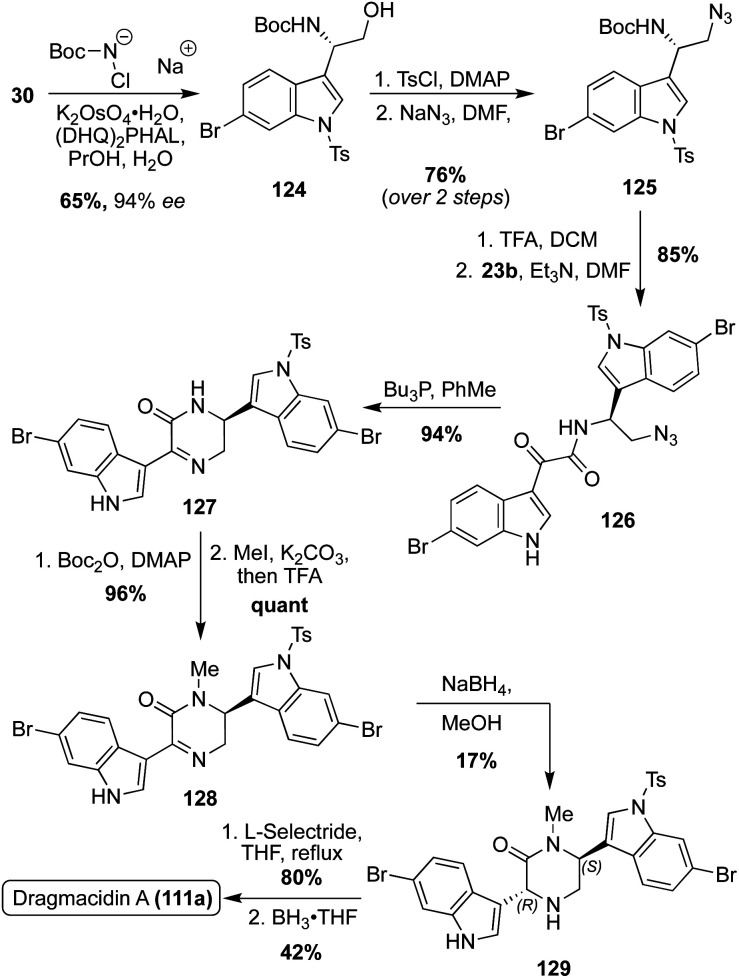
First total synthesis of optically active Dragmacidin (111).

Optically active Dragmacidin A (111a) was synthesized *via* a Sharpless asymmetric aminohydroxylation of 6-bromo-3-vinylindole (30) to access 124 in 65% yield and 94% enantiomeric excess, as shown in Scheme 27. The hydroxyl group of 124 was then tosylated and subsequently substituted by an azido group in high yield over two steps. The resulting amino azide intermediate (125) was then deprotected and acylated with 23b, followed by reduction of the azide and subsequent cyclization to afford 127. After Boc-protection, 127 was selectively and quantitatively methylated and subsequently deprotected to afford 128. The reduction of intermediate 128 proved to be problematic, as it was nonselective. In fact, the desired isomer (129) was the minor product in just 17% isolated yield (82% of *cis* isomer isolated). Despite this low yield, 129 was de-tosylated *via*l-selectride and the piperazinone was reduced to the desired piperazine core to access (2*S*, 5*R*)-Dragmacidin A (111a) in 42% yield over two steps.^81^

**Scheme 28:**
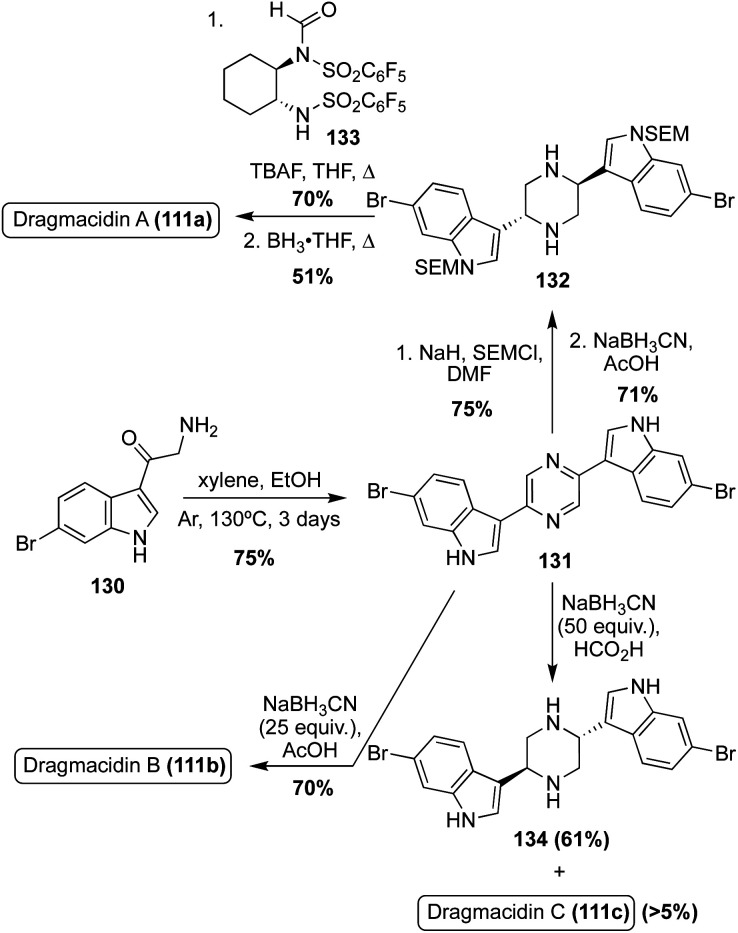
Total syntheses of Dragmacidins A–C (111a–c).

An expedited method of synthesizing optically active Dragmacidin A (111a) was developed in which a bis-indolylpyrazine intermediate (131) was protected and subsequently reduced to the corresponding piperazine intermediate (132) in high yields, as shown in Scheme 28. Then, de-symmetrization of the piperazine (132) was completed *via* enantioselective formylation using a chiral formylating reagent. This method ultimately proved to be only moderately stereoselective, affording the dextrorotatory isomer of Dragmacidin A (111a) in 44% enantiomeric excess after transformation of the aldehyde to a methyl group and subsequent deprotection.^82^ Using a similar reductive approach to that used on the bis-indolylpyrazine intermediate (131), an efficient synthesis of Dragmacidin B (111b) was achieved in 53% overall yield over two steps, as shown in Scheme 28. It should be noted that this was not an efficient method for synthesizing Dragmacidin C (111c), as the undesired isomer predominated and less than 5% yield of Dragmacidin C (111c) was isolated.^83,84^

**Scheme 29:**
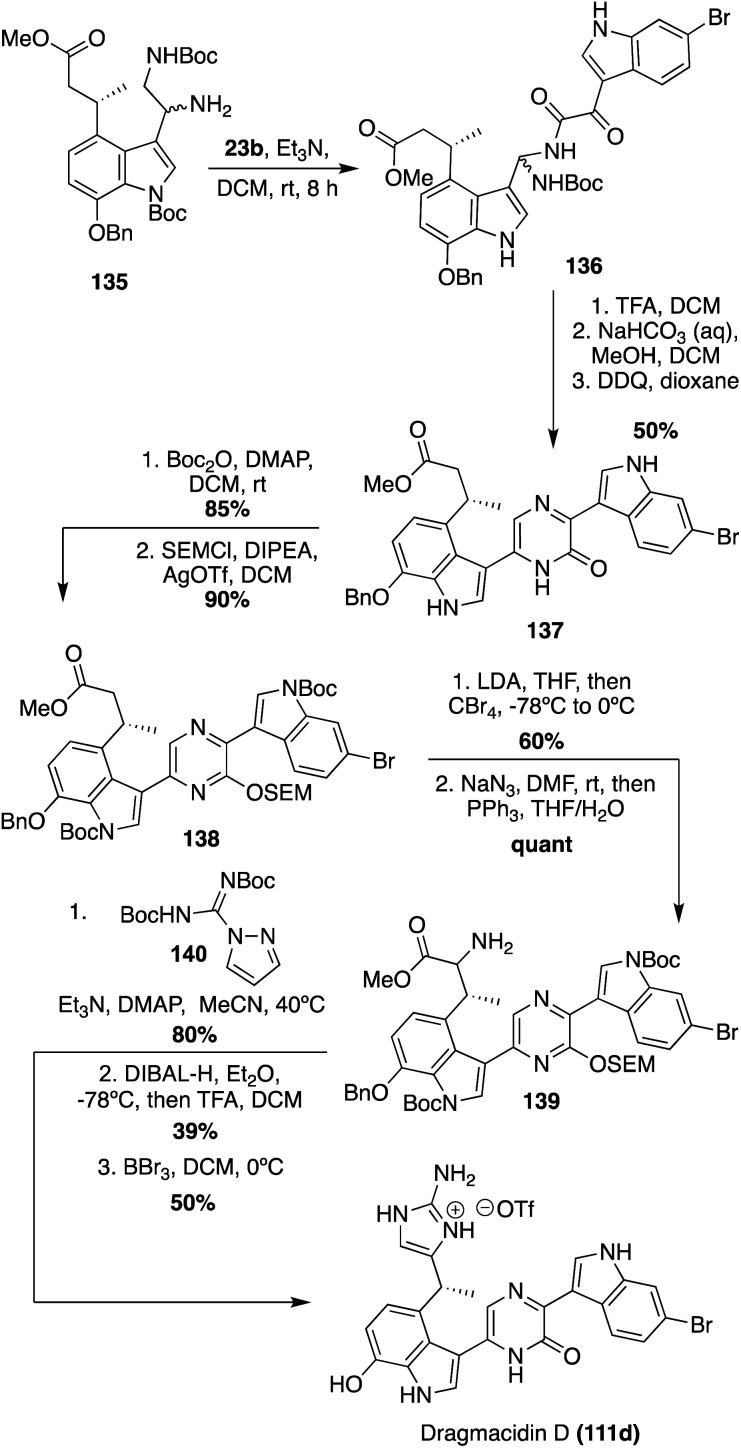
First total synthesis of Dragmacidin D (111d).

Recently, the first total synthesis of the more structurally complex Dragmacidin D (111d) was completed.^85^ First, the iodinated indolic diamine intermediate (135) was synthesized in 11 linear steps from commercially available starting materials. Here, the desired stereoisomer of 135 was accessed *via* the use of Evan's oxazolidinone chiral auxiliary ((*S*)-3-acryloyl-4-phenyloxazolidin-2-one) and column chromatography.^85^ After the key diamine intermediate (135) was synthesized, it was condensed with 6-bromo-1*H*-indole-3-oxo-acyl chloride and subsequently cyclized and oxidized to access 2-piperazinone intermediate (137) in 50% yield over multiple steps, as shown in Scheme 29. Then, 137 was protected to allow for selective α-bromination, followed by a Staudinger reaction to access the α-amino ester (139) in high yields. Treatment of this α-amino ester (139) with pyrazole-1-carboxamidine afforded a guanidine intermediate, which underwent subsequent reduction and cyclization to construct the 2-aminoimidazole moiety of Dragmacidin D in good yields. Deprotection then afforded Dragmacidin D (111d) in 50% yield. This synthesis revised the earlier stereochemical assignment that was based on biosynthetic considerations, assigning the absolute configuration as (*R*)-(+)-Dregmacidin D (111d). Furthermore, chiral HPLC-DAD methodology was developed and utilized to confirm, for the first time, that naturally occurring Dragmacidin D (111d) was isolated as either a racemate or a scalemic mixture (39% ee), which has prompted questions regarding the absolute configurations of Dragmacidins D-F (111d–f).^85^

#### 2.4.2. Hamacanthins

**Fig. 11:**
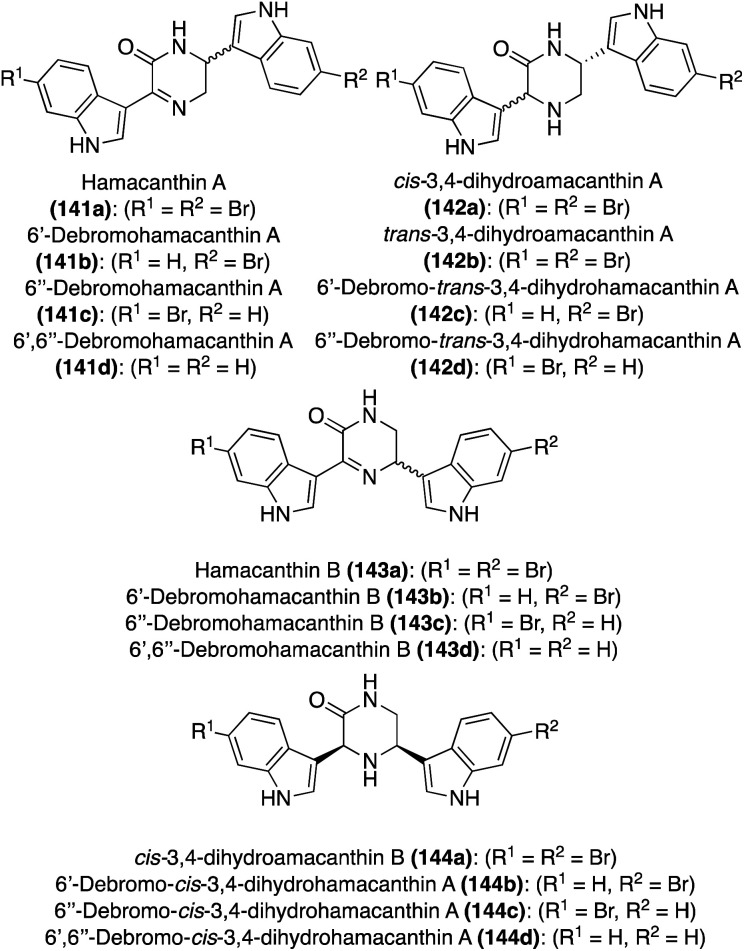
Chemical structures of Hamacanthins A–B (141a and 143a), Dihydrohamacanthins A–B (142a and 144a), and related debrominated natural products.

Another class of bis-indole alkaloids that are structurally related to the Dragmacidins are the Hamacanthins (141a–d, 142a–d, 143a–d, 144a–d), which contain a 5,6-dihydro-2-pyridone or 2-piperazinone core connecting the two indole moieties, as shown in Fig. 11. The first of these natural products to be isolated were Hamacanthin A (141a) and Hamacanthin B (143a), which were isolated from *Hamacantha* marine sponge in 1994 by Gunasekera, *et. al.* These natural products were found to display antifungal activity.^86^ In more recent years, the de-bromo and dihydro Hamacanthin analogues (141b–d, 142a–d, 143b–d, 144a–d) have been isolated from various natural marine sources, as shown in Table 2. The configurations of these natural products are also indicated in Table 2, and many were later confirmed *via* total syntheses. Despite their structural similarities, the configurations of these Hamacanthins vary. For example, Hamacanthin A (141a) exists as the (*S*)-isomer, while its analogous mono de-bromo natural products (141b and 141c) exist as the (*R*)-isomers, and its bis de-bromo analogue (141d) exists as the (*S*)-isomer (Table 2). Overall, natural products of the Hamacanthin class (141a–d, 142a–d, 143a–d, 143a–d) have been shown to display antifungal, antibacterial, and cytotoxic activity.^86^

**Table 2:** Hamacanthin class isolation data^2,7,8,12,86,89^

Compound	Configuration	Natural source
141a	*S*	*Hamacantha*, *Spongosorites*, *Disodermia calyx*
141b	*R*	*Spongosorites*
141c	*R*	*Spongosorites*, *Discodermia calyx*
141d	*S*	*Spongosorites*
143a	*S*	*Hamacantha*, *Spongosorites*, *Disodermia calyx*
143b	*R*	*Spongosorites*, *Discodermia calyx*
143c	*R*	*Spongosorites*
143d	*R*	*Spongosorites*
142a	3*R*, 6*R*	*Rhaphizia lacazei*
142b	3*S*, 6*R*	*Rhaphizia lacazei*, *Spongosorites*
142c	3*S*, 6*R*	*Rhaphizia lacazei*, *Spongosorites*
142d	3*S*, 6*R*	*Rhaphizia lacazei*, *Spongosorites*
144a	3*S*, 5*R*	*Rhaphizia lacazei*, *Spongosorites*, *Discodermia calyx*
144b	3*S*, 5*R*	*Rhaphizia lacazei*, *Spongosorites*
144c	3*S*, 5*R*	*Rhaphizia lacazei*, *Spongosorites*
144d	3*S*, 5*R*	*Spongosorites*

**Scheme 30:**
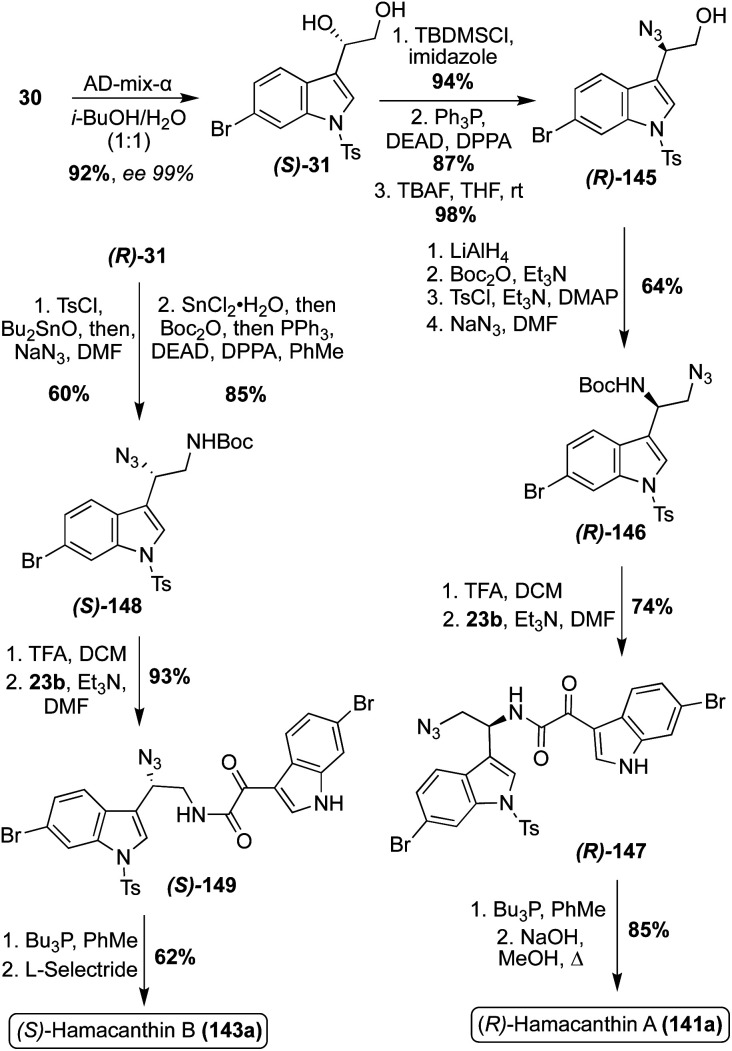
Enantioselective total syntheses of Hamacanthins A and B (141a and 143a).

The first enantioselective total synthesis of the unnatural (*R*)-Hamacanthin A (141a) was completed in 2001 by Jiang, *et. al. via* the coupling of a 3-indolyl-α-oxo-acetyl chloride intermediate (23b) and 3-indolyl azidoethylamine intermediate (*R*)-(146), prior to an intramolecular aza-Wittig type cyclization to access (*R*)-Hamacanthin A (141a) in high yields, as shown in Scheme 30.^87^ The 3-indolyl azidoethylamine intermediate was synthesized in four steps from *N*-tosylated-6-bromo-3-vinyl indole in high yields. The stereocenter of (141a) was established *via* a Sharpless asymmetric dihydroxylation reaction, followed by a stereospecific azidation. In spectroscopic comparison of the naturally isolated Hamacanthin A (141a) and the synthesized (*R*)-Hamacanthin A (141a), it was confirmed that the natural Hamacanthin A (141a) existed as the (*S*)-isomer.^87,88^

A year later, the same research team completed the first enantioselective total synthesis of Hamacanthin B (143a). As shown in Scheme 30, this synthesis was completed *via* a similar approach as was previously discussed for (*R*)-Hamacanthin A (141a). Considering the established (*S*)-configuration of (141a), (*S*)-Hamacanthin B (143a) was synthesized. When compared to the naturally isolated Hamacanthin B (143a), the synthesized (*S*)-143a confirmed the configuration of the natural 143a as the (*S*)-isomer.^88^

**Scheme 31:**
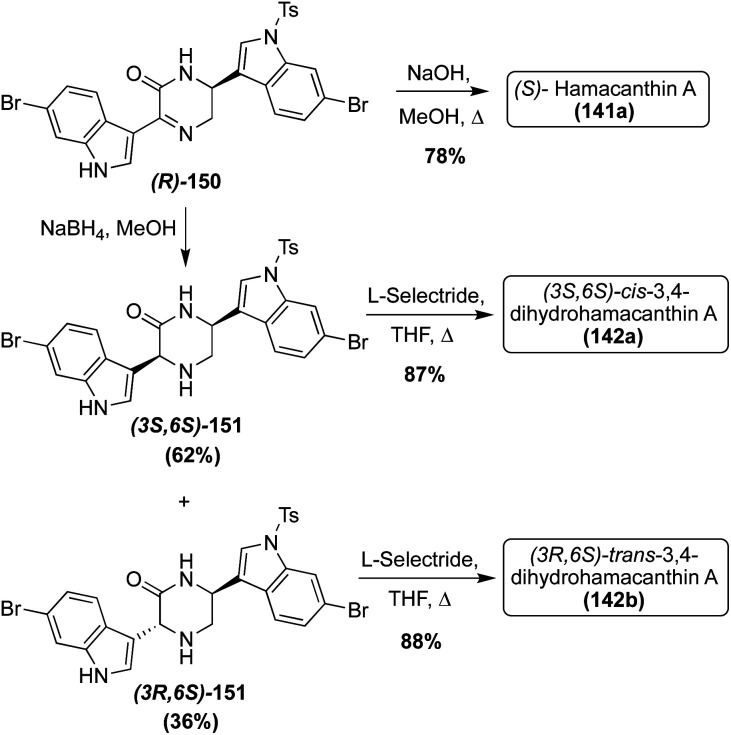
Enantioselective total syntheses of Hamacanthin A (141a) and Dihydrohamacanthin A–B (142a–b).

With the elucidation of the absolute configuration of (*S*)-Hamacanthin A (141a), the first enantioselective total synthesis of the desired (*S*)-Hamacanthin A (141a) was completed *via* deprotection and simultaneous inversion of the stereocenter of (*R*)-150 to access (*S*)-141a in 78% yield, as shown in Scheme 31. This allowed for additional confirmation that this was the correct configuration of the naturally occurring (*S*)-Hamacanthin A (141a). The synthetic approach toward (*R*)-150 was previously discussed in the Dragmacidin section (Scheme 27).^81^ Also, using intermediate (*R*)-150, the first total syntheses of the unnatural (3*S*, 6*S*)-*cis*-3,4-dihydrohamacanthin A (142a) and (3*R*, 6*S*)-*trans*-3,4-dihydrohamacanthin A (142b) were both completed. The reduction of (*R*)-150 afforded 62% yield of (3*S*, 6*S*)-*cis*-151 and 36% yield of (3*R*, 6*S*)-*trans*-151. These 2-piperidone intermediates (151) were then deprotected *via*l-selectride to access (3*S*, 6*S*)-*cis*-3,4-dihydrohamacanthin A (142a) and (3*R*, 6*S*)-*trans*-3,4-dihydrohamacanthin A (142b) in 87% and 88% yields, respectively (Scheme 31). *Via* comparison of the naturally isolated *cis*-3,4-dihydrohamacanthin A (142a) and *trans*-3,4-dihydrohamacanthin A (142b) with the synthesized (3*S*, 6*S*)-*cis*-142a and (3*S*, 6*S*)-*trans*-142b, it was found that the configurations of these synthesized natural products did not match that of the isolated optical rotation data. Therefore, it was concluded that the absolute configurations of *cis*-3,4-dihydrohamacanthin A (142a) and *cis*-3,4-dihydrohamacanthin A (142a) are (3*R*, 6*R*) and (3*S*, 6*R*), respectively.^81^ Additionally, racemic *cis*- and *trans*-3,4-dihydrohamacanthins can be accessed, albeit in low yields, *via* partial reduction of cyclized dipeptides (121 and 123), as was also discussed in the Dragmacidin section (Scheme 26).^72^

**Scheme 32:**
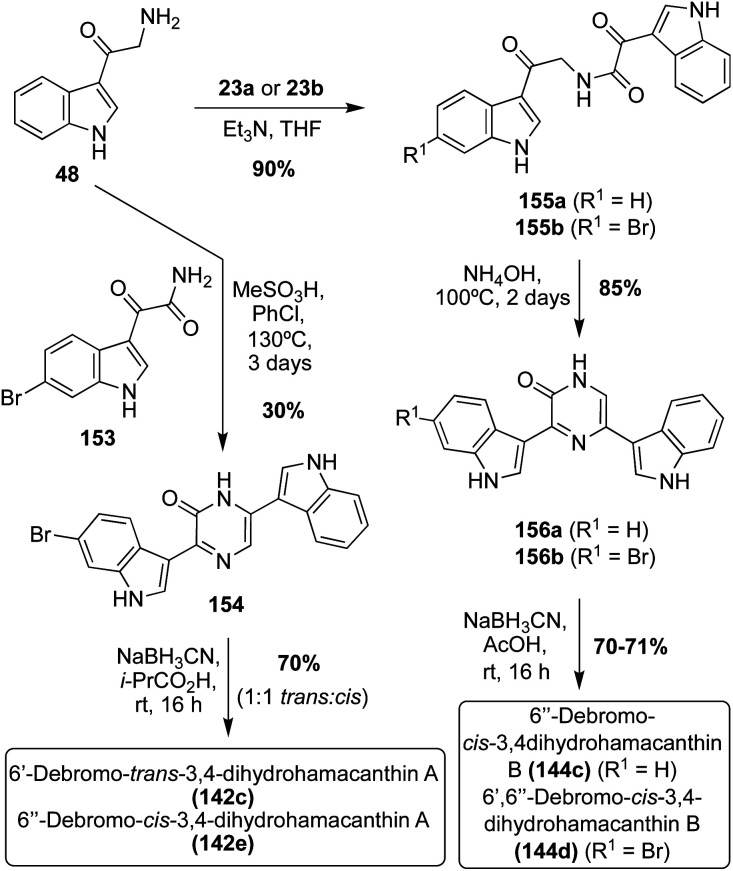
Enantioselective total syntheses of Debromo-Dihydrohamacanthins (142c) and (144c–d).

The first total syntheses of racemic 6′-debromo-*trans*-3,4-dihydrohamacanthin A (142c) and the unnatural 6′′-debromo-*cis*-3,4-dihydrohamacanthin A (142e) were carried out *via* the condensation and subsequent cyclization of an indolic α-amino ketone intermediate (48) with an indolic keto–amide intermediate (153). After formation of this bis-indolylpyrazinone intermediate, it underwent reduction to access 6′-debromo-*trans*-3,4-dihydrohamacanthin A (142c) and the unnatural 6′′-debromo-*cis*-3,4-dihydrohamacanthin A (142e) in 70% yield as a 1 : 1 mixture (Scheme 32). While it was not enantioselective, this method proved to be a very expeditious synthesis of these natural products.^90^ This method also allowed for the first racemic total synthesis of 6′′-debromo-*cis*-3,4-dihydrohamacanthin B (144c) and 6′,6′′-debromo-*cis*-3,4-dihydrohamacanthin B (144d). As shown in Scheme 32, this method was adapted to achieve the 3,5-disubstituted cyclized product by condensing and cyclizing an indolic oxo-acyl chloride intermediate (23a–b) with an indolic α-amino ketone intermediate (48). In addition to being an efficient and high yielding route, this method was selective for the desired *cis*-isomers of 6′′-debromo-*cis*-3,4-dihydrohamacanthin B (144c) and 6′,6′′-debromo-*cis*-3,4-dihydrohamacanthin B (144d), respectively.^90^

**Scheme 33:**
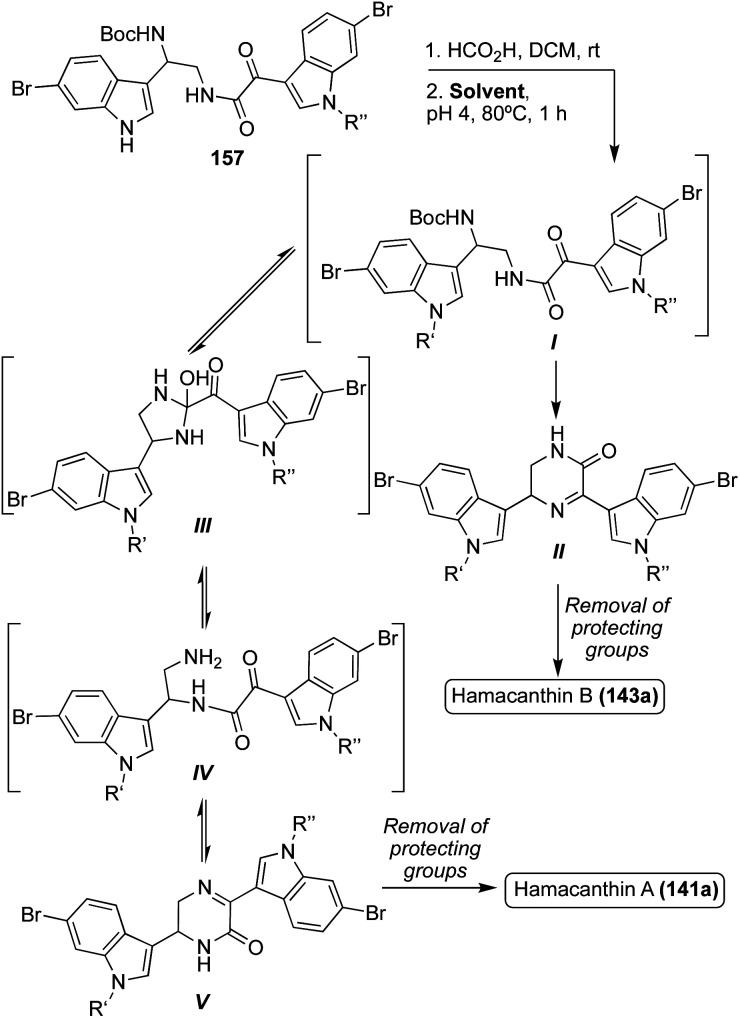
Total syntheses of Hamacanthins A and B (141a) (143a).

A similar bio-mimetic cyclization of a ketoamide (157) was proven efficient for the synthesis of both racemic Hamacanthin A (141a) and Hamacanthin B (143a). As shown in Scheme 33, to access Hamacanthin B (143a), nucleophilic attack of the primary amine on the indolic (C3) ketone and subsequent dehydration led to intermediate (II), which was deprotected to access 143a. On the other hand, nucleophilic attack of the primary amine on the amide carbonyl to form a five-membered ring intermediate (III), followed by a ring opening that resulted in a rearrangement of the original ketoamide to intermediate (IV). Cyclization of intermediate (IV) then led to Hamacanthin A (141a) after removal of protecting groups.^90^ Through mechanistic study, it was determined that the ratio of 141a : 143a was heavily dependent on the identity of the solvent, and the protecting groups (R′ and R′′), as indicated by the results shown in Table 3. In this case, more polar, protic solvents, such as ethanol, led to predominant formation of intermediate (II) while more non-polar solvents, such as dichloroethane (DCE), led to predominant formation of intermediate (V). In addition, the presence of stronger electron-withdrawing groups, such as a tosyl group, on the indole nitrogen led to predominant formation of intermediate (II) over intermediate (V) due to the resulting increased electrophilicity of the carbonyl group adjacent to the indole, making nucleophilic attack on this carbonyl preferable.^90^

**Table 3:** Cyclization conditions of keto–amide (157)

R′	R′′	Solvent	Yield (V)	Yield (II)
Ts	H	DCE	42%	35%
Ts	H	1,4-Dioxane	30%	38%
Ts	H	EtOH	15%	75%
Ts	H	DMF	7%	46%
Ts	Ac	DCE	42%	55%
Ts	Ts	DCE	18%	68%

**Scheme 34:**
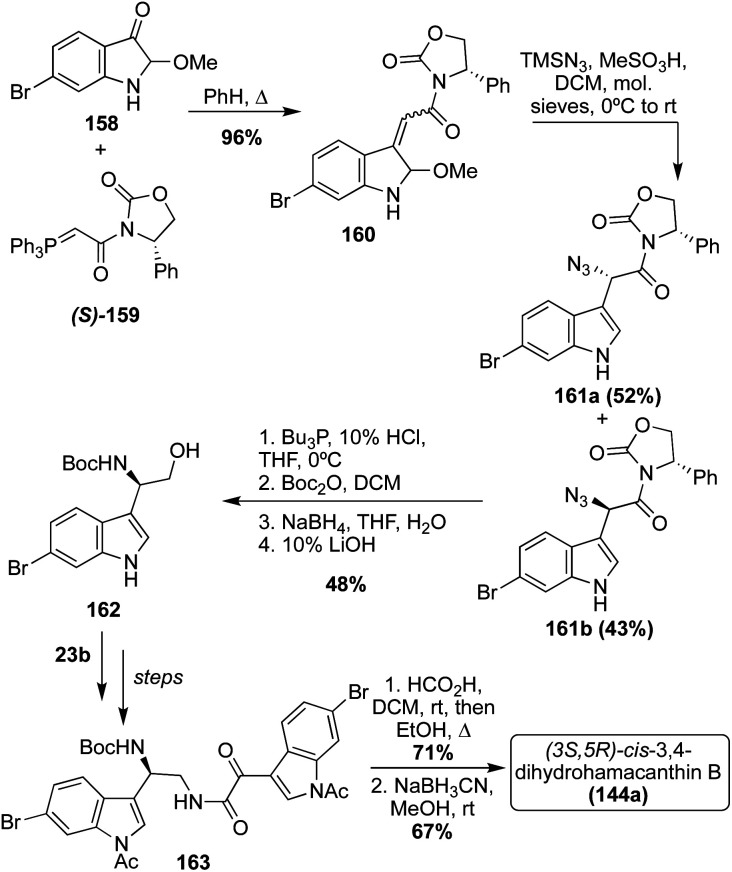
First enantioselective total synthesis of Dihydrohamacanthin B (144a).

In 2007, along with enantioselective total synthesis of (*S*)-Hamacanthins A and B (141a and 143a), the first enantioselective total synthesis of *cis*-3,4-dihydrohamacanthin B (144a) was also completed.^91^ As shown in Scheme 34, this synthesis proceeded *via* the condensation of an indolic amino–alcohol intermediate (162) and an indolic oxo-acyl chloride intermediate (23b) to access a similar keto–amide intermediate (163), as was previously discussed. Subsequent cyclization of the keto–amide intermediate (163) and stereospecific reduction afforded (3*S*-,5*R*)-*cis*-3,4-dihydrohamacanthin B (144a) in high yields. Here, the stereochemistry was set *via* the installation of an (*S*)-phenyloxazolidone chiral auxiliary (*S*)-(159).^91^

**Scheme 35:**
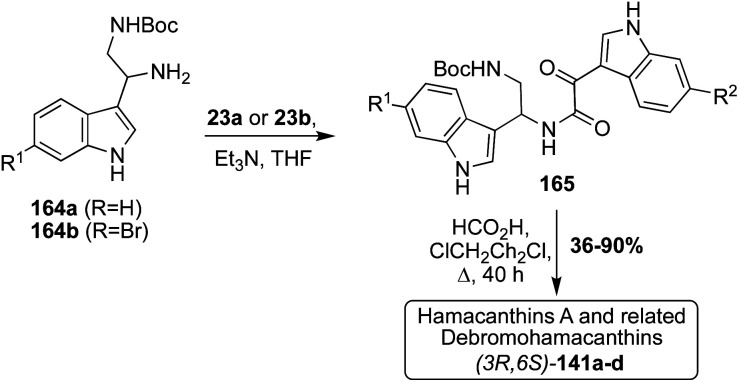
Total syntheses of Hamacanthin A (141a) and related Debromohamacanthins (141b–d).

In a slightly adapted, but very similar condensation of an indolic diamine intermediate (164) and an indolic oxo-acyl chloride intermediate (23a–b) and following cyclization, racemic Hamacanthin A (141a) and its related Debromohamacanthins (141b–d) were accessed in moderate to high yields, as shown in Scheme 35.^92^ The indolic diamine intermediates (164a–b) were synthesized using the same method discussed previously in the Spongotine section (Scheme 5). At present, several debromo analogues of Hamacanthin B and Dihydrohamacanthin B have yet to be accessed *via* total synthesis.

#### 2.4.3. Fellutanines

**Fig. 12:**
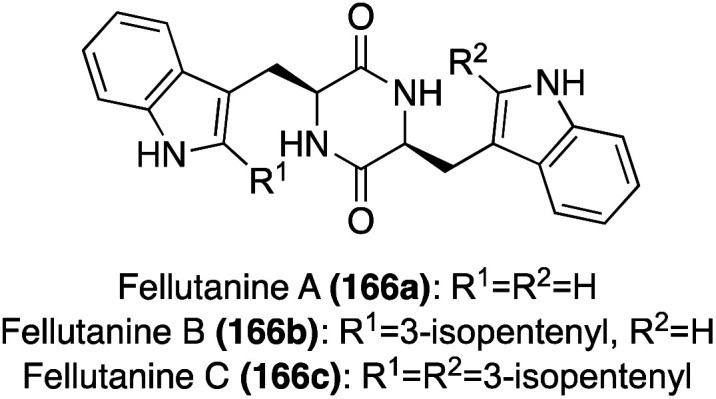
Chemical structures of Fellutanines A–C (166a–c).

Fellutanines A–C (166a–c) were first isolated in 2000 by Kozlovsky *et. al.* from the fungi *Penicillium fellutanum*. The 3,6-di-substituted-bis-indolyl-piperazine-2,5-dione structures of these natural products was elucidated *via* spectroscopic methods (Fig. 12). In addition, it was discovered that Fellutanine B (166b) contained an isopentenyl group on the 2-position of one of the indole moieties and Fellutanine C (166c) contained two isopentenyl substituents, one on the 2-position of each indole moiety.^93^ The configuration of these Fellutanines A–C (166a–c) was initially reported as the *cis*-isomer, but this structure was later revised to the correct *trans*-isomer of the piperazine-2,5-dione core.^93^ Much of the biological activity of these compounds is unknown, though some studies have shown they possess antibacterial activity, but no significant cytotoxic activity.^93,94^

**Scheme 36:**
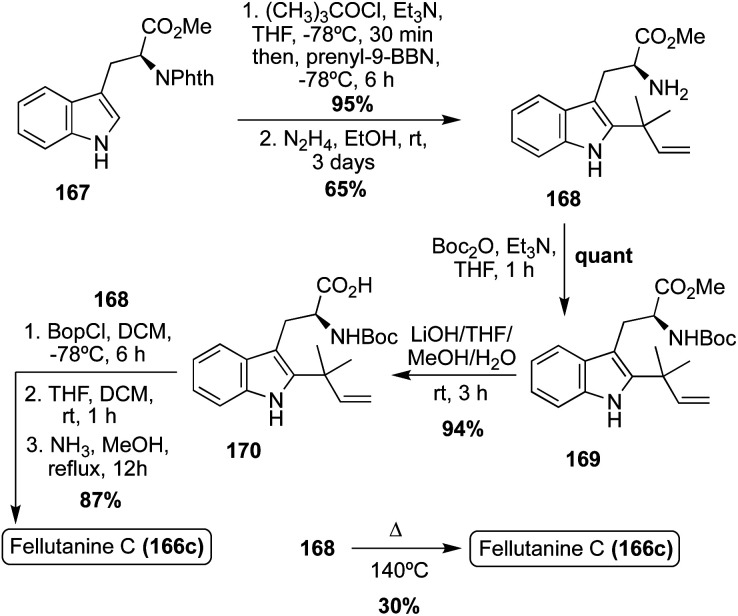
First total synthesis of Fellutanine C (166c).

Interestingly, the first total synthesis of Fellutanine C (166c) was completed before its first isolation, as it was an intermediate that was accessed in the 1995 total synthesis of another related fused bicyclic natural product, Gypsetin.^95^ As is shown in Scheme 36, this total synthesis began with indolic C-2 reverse prenylation of *N*-phthaloyl-l-tryptophan methyl ester (167) *via* prenyl-9-BBN in high yield, followed by hydrazinolysis and subsequent Boc-protection and saponification to access intermediate (170) in high yields. Then, 170 and 168 were coupled to access the desired *cis*-Fellutanine C (166c) in a very high 87% yield over three steps.^95^

In a similar approach using l-tryptophan analogues as starting materials, Fellutanine C (166c) was synthesized *via* the same intermediate (168) used previously, though here undergoing an expeditious dimerization and cyclization *via* pyrolysis conditions to access *cis*-Fellutanine C (166c) in 30% yield.^96^ This proved to be an advantageous process in terms of step count by saving four steps compared to the previous synthesis, yet the yield decreased significantly in spite of this.

**Scheme 37:**
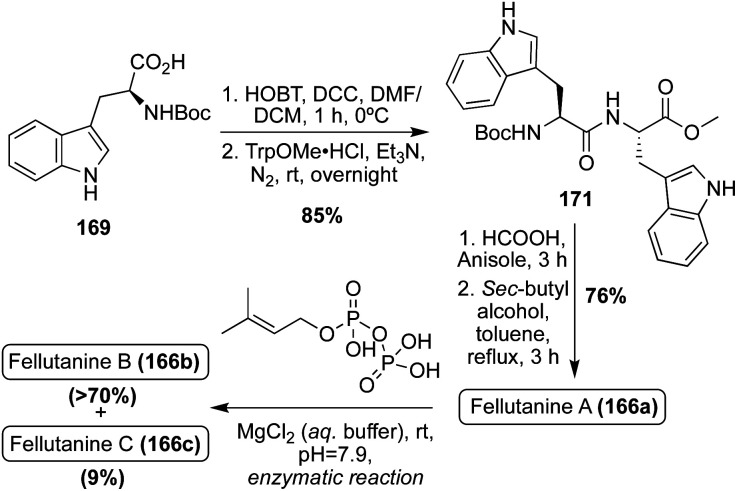
First total syntheses of Fellutanine A–B (166a–b).

The first total synthesis of Fellutanine A (166a) was completed in 2008 in a similar method to that of Fellutanine C (166c). Here, a tryptophan analogue (169) and TrpOMe·HCl were condensed and subsequently cyclized to access the desired *cis*-Fellutanine A (166a) in 76% yield (Scheme 37).^97^

In addition to the previously discussed synthetic methods of accessing these Fellutanine natural products, many enzymatic biosynthetic methods have also been utilized over the years, using l-tryptophan as the starting material to access these molecules^98,99^ Recently, a semi-total synthesis was developed and conducted to achieve all three natural products, Fellutanines A–C (166a–c), *via* the same route. As shown in Scheme 37, the same condensation and subsequent cyclization of tryptophan derivatives (169) and TrpOMe·HCl was conducted to access *cis*-Fellutanine A (166a) in 76% yield. Then, the enzymatic C2-reverse-prenylation of indole was performed *via* DMAPP prenyltransferase and aqueous MgCl_2_ under slightly basic conditions, to access the mono-reverse prenylated *cis*-Fellutanine B (166b) as the major product in >70% yield. It is noteworthy to mention that, while not the major product, *cis*-Fellutanine C (166c) was also accessed *via* this method in 9% yield. The most significant achievement of this work was the first total synthesis of Fellutanine B (166b) and the development of a late stage selective reverse prenylation methodology of indole. It was also a significant that all three Fellutanines A–C (166a–c) could be accessed *via* this same route (Scheme 37).^100^

### 2.5 Pyridine and diazine linker moieties

#### 2.5.1. Hyrtinadine/Alocasin/Scalaridine

**Fig. 13:**
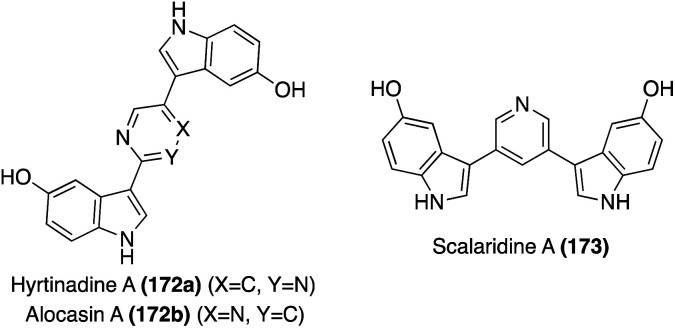
Chemical structures of Hyrtinadine A (172a), Alocasin A (172b), and Scalaridine A (173).

Three structurally related bis-indole natural products with heterocyclic aromatic linker moieties were discovered between 2007 and 2013. The first of these was Hyrtinadine, A (172a), which was isolated in 2007 by Endo, *et. al.* from the Okinawan marine sponge *Hyrtios* sp.^101^ This bis-indolyl-2,5-di-substituted pyrimidine structure was elucidated *via* spectroscopic methods (Fig. 13). Hyrtiniadine A (172a) was also found to be highly cytotoxic towards murine Leukemia L1210 and human epidermoid carcinoma KB cell lines.^101^ The next of these related natural products to be discovered was Alocasin A (172b), which was isolated in 2012 by Zhu, *et. al.* from the dried rhizomes of the herbaceous plant *Alocasia macrorrhiza*.^102^ Alocasin A (172b) was reportedly the first heterocycle-linked bis-indole alkaloid isolated from a terrestrial source. As shown in Fig. 13, the chemical structure of (172b) has remarkable structural similarity to that of Hyrtinidine A (172a). However, the two indole moieties of Alocasin A (172b) are connected by a pyrazine linker moiety, rather than the pyrimidine of (172a). Much is unknown regarding the biological activity of Alocasin A (172b); however, it has been shown to display weak antiproliferative activity against Hep-2 and Hep-G2 cell lines.^102^ The most recent of these to be discovered was Scalaridine A (173), which was isolated in 2013 by Lee *et. al*. from the marine sponge *Scalarispongia* sp., along with the previously discussed Hyrtinidine A (172a).^103^ This natural product (173) was determined to have a bis-indolyl-2,5-di-substituted pyridine structure. Interestingly, this is the only bis-indole alkaloid of its kind to contain a pyridine linker moiety. Much is also unknown regarding the biological activity of Scalaridine A (173), but much like its two aforementioned structural relatives, it also displayed significant cytotoxicity.^103^

**Scheme 38:**
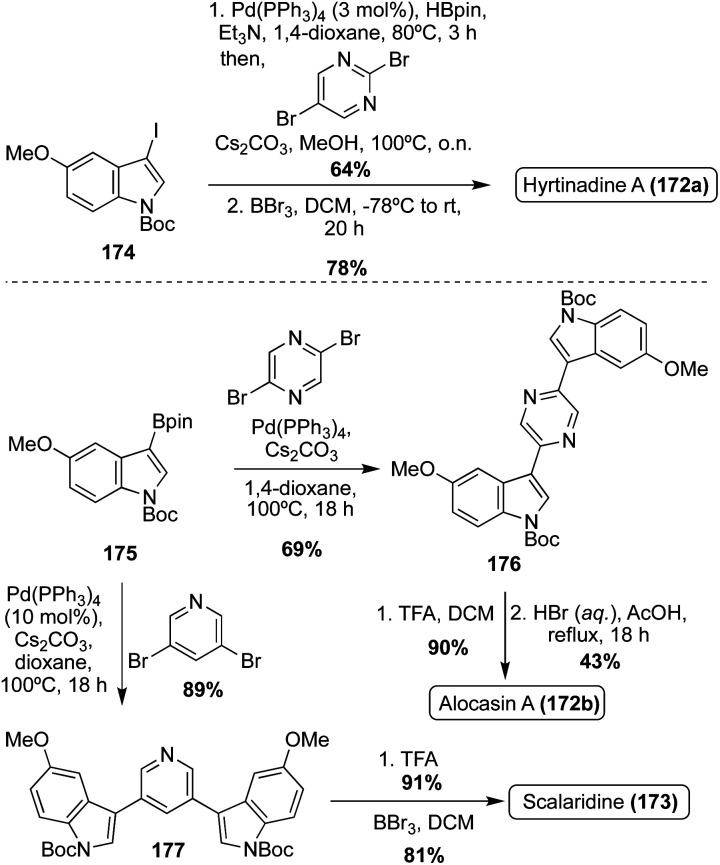
First total syntheses of Hyrtinadine A (172a), Alocasin A (172b), and Scalaridine A (173).

The first of these natural products to be accessed *via* total synthesis was Hyrtinadine A (172b) in 2011, by Müller, *et. al.* As shown in Scheme 38, the first total synthesis of Hyrtinadine A (173) was achieved *via* sequential palladium-catalyzed Masuda borylation and double Suzuki cross-coupling reactions to install the two indole moieties on the 2- and 5-positions of the pyrimidine core. Subsequent de-methylation *via* BBr_3_ achieved Hyrtinadine A (172a) in 78% yield.^104^

In 2015, the first total syntheses of Alocasin A (172b) and Scalaridine A (173) were completed *via* a similar borylation and subsequent double Suzuki coupling route as was used in the first total synthesis of Hyrtinadine A (172a), followed by de-methylation of the indolic hydroxyl groups to access Alocasin A (172b) and Scalaridine A (173) in high yields.^105,106^

**Scheme 39:**
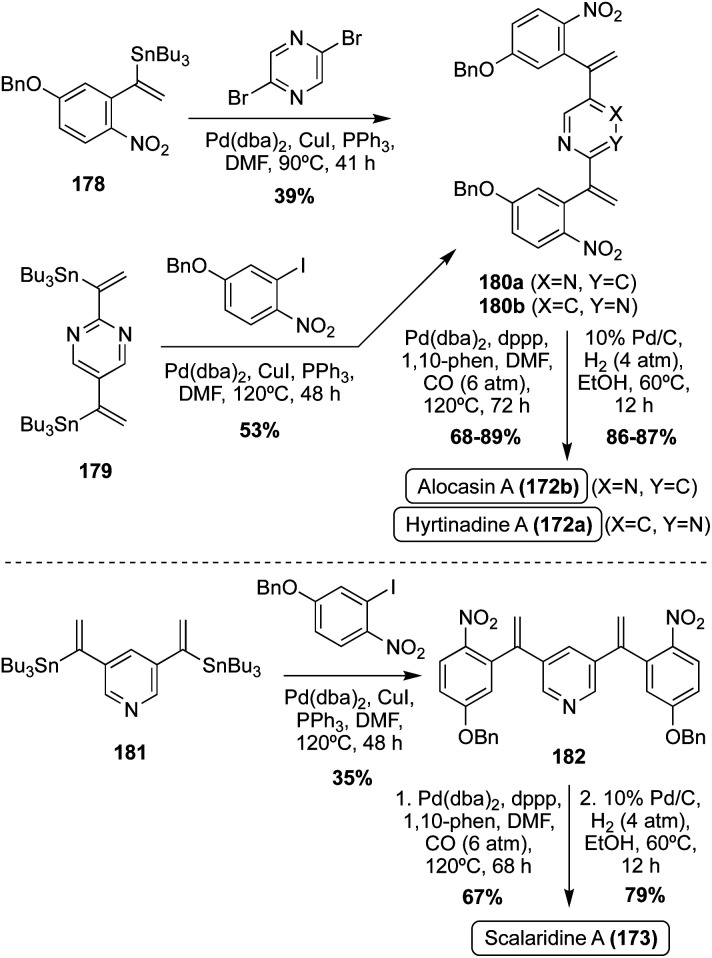
Total syntheses of Hyrtinadine A (172a), Alocasin A (172b), and Scalaridine A (173).

One year later, a different approach to the total synthesis of all three natural products (172a, 172b, and 173), was conducted *via* successive palladium-catalyzed reactions; a Kosugi–Migita–Stille cross coupling reaction to install the aromatic substituents on the heterocyclic linker, a reductive N-heterocyclization to form the indole moieties, and a final hydrogenolysis to access 172a, 172b, and 173 in high yields, as shown in Scheme 39.^107^

As can be concluded from the previously discussed syntheses of Hyrtinidine A (172a), Alocasin A (172b), and Scalaridine A (173), the common method of synthesis for these natural products is the use of palladium-catalyzed cross-coupling reactions to install the indole moieties on the aromatic N-heterocyclic cores.

**Scheme 40:**
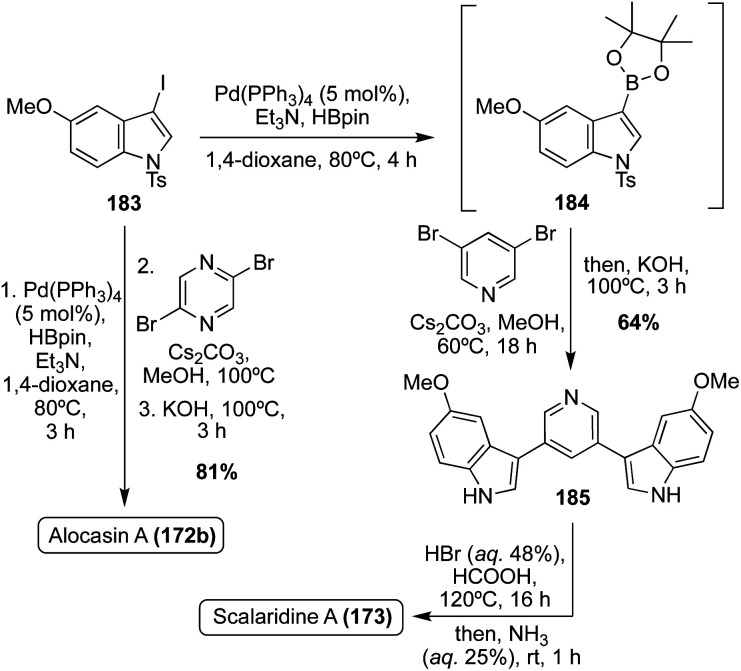
Total syntheses of Alocasin A (172b) and Scalaridine A (173).

In recent years, additional total syntheses of 172a–b and 173 have been reported using similar palladium-catalyzed cross coupling approaches.^108–110^ A couple examples of these are shown in Scheme 40, such as the 2022 total synthesis of Scalaridine A (173) and the significantly expedited one-pot approach using a similar palladium-catalyzed methodology in the total synthesis of Alocasin A (172b). The latter achieved Alocasin A (172b) in 81% from the iodoindole intermediate (183), using a three phase, one-pot method.^111^

## 3. Tris-indole alkaloids

Compared to their previously discussed bis-indole alkaloid counterparts, tris-indole alkaloids are rarer and more elusive in nature. However, in recent years, discovery and synthesis of these natural products has become more prevalent.

### 3.1 Aromatic N-heterocyclic linker moieties

#### 3.1.1. Gelliusines

**Fig. 14:**
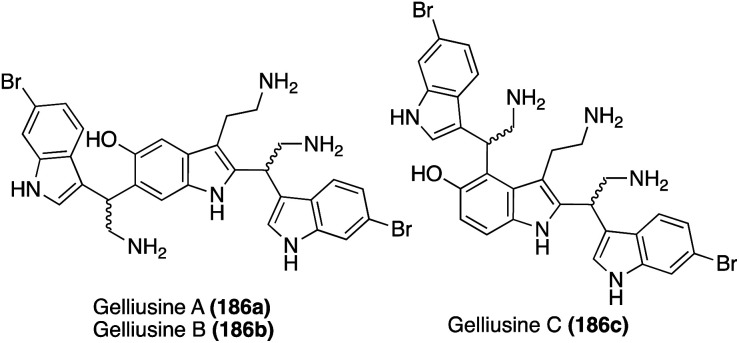
Chemical structures of Gelliusines A–C (186a–c).

Gelliusines A (186a) and B (186b) were first isolated as enantiomeric pairs in 1994 by Bifulco, *et. al.* from the deep-water marine sponge *Gellius* sp. (also known as *Orina* sp).^112^ The unique tris-indole chemical structure of these natural products, in which an indole core connects two additional indole moieties at the C-2 and C-6 positions, was elucidated *via* spectroscopic methods, as shown in Fig. 14. In addition, Gelliusines A (186a) and B (186b) were determined to also contain three amine substituents. These amine substituents were of particular interest as it is hypothesized that they could contribute to favorable water-solubility of these natural products, which could be beneficial in bioactive applications. The absolute stereochemistry of the chiral centers of 186a and 186b was inconclusive *via* obtained data and, because Gelliusines A (186a) and B (186b) have yet to be accessed *via* total synthesis, their absolute configurations remain unknown. In terms of their biological activity, Gelliusines A (186a) and B (186b) were found to display cytotoxic activity, as well as anti-serotonin activity. The latter activity is unsurprising considering the structural similarities of Gelliusines A (186a) and B (186b) and serotonin.^112^

One year later, an additional structurally similar natural product of this class, Gelliusine C (186c) was isolated by the same group from the deep-water marine sponge *Gellius* sp. (also known as *Orina* sp). As shown in Fig. 14, the chemical structure of Gelliusine C (186c) was determined to be a similar tris-indole alkaloid containing in indole core connecting two additional indole moieties, as well as containing three amine substituents. The main difference was, instead of the 2,6-substituted indole core of 186a–b, the indole core of Gelliusine C (186c) had the two additional indole moieties connected at the C2 and C4 positions. Similar to 186a–b, the absolute configuration of Gelliusine C (186c) was inconclusive *via* obtained data and, because Gelliusines C (186c) have yet to be accessed *via* total synthesis, its absolute configuration also remains unknown. In terms of their biological activity, Gelliusine C (186c) exhibited cytotoxic activity, as well as anti-serotonin activity.^113^

#### 3.1.2. Tricepyridinium

**Fig. 15:**
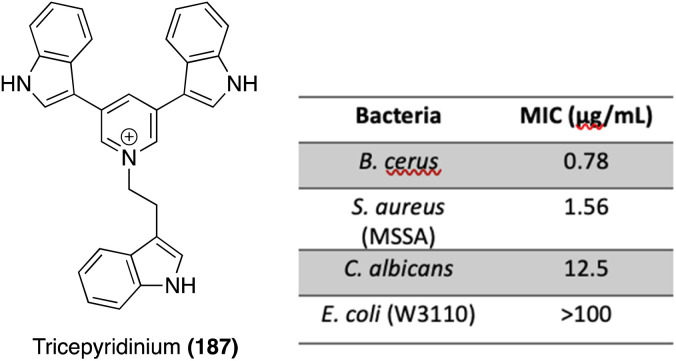
Chemical structure of Tricepyridinium (187) and its related biological activity data.

Tricepyridinium (187) is a tris-indole alkaloid with a quaternary pyridinium core that was first isolated in 2017 by Abe *et. al.* from a culture of *Escheichia coli* clone incorporating metagenomic libraries from the marine sponge *Discodermia calyx*. The 1,3,5-tri-substituted pyridinium core connecting the three indole moieties of Tricepyridinium (187) was elucidated *via* spectroscopic methods, as shown in Fig. 15.^114^ Tricepyridinium (187) was found to display potent antibacterial activity, which is summarized in Fig. 15. Several bis-indole analogues of Tricepyridinium (187) were also synthesized and tested in this initial exploration of biological activity and 187 displayed more potent antibacterial activity than any of these analogues. Therefore, it was concluded that all three indole moieties of 187 were necessary for its potent antibacterial activity. In contrast, 187 did not display any cytotoxic activity against murine leukemic P388 cell line, while a bis-indole analogue of 187 lacking the indole moiety on the pyridinium nitrogen exhibited significant cytotoxic activity against this same cell line. This indicated that the cytotoxic activity of Tricepyridinium (187) was improved *via* removal of the indole in this position.^114^

**Scheme 41:**
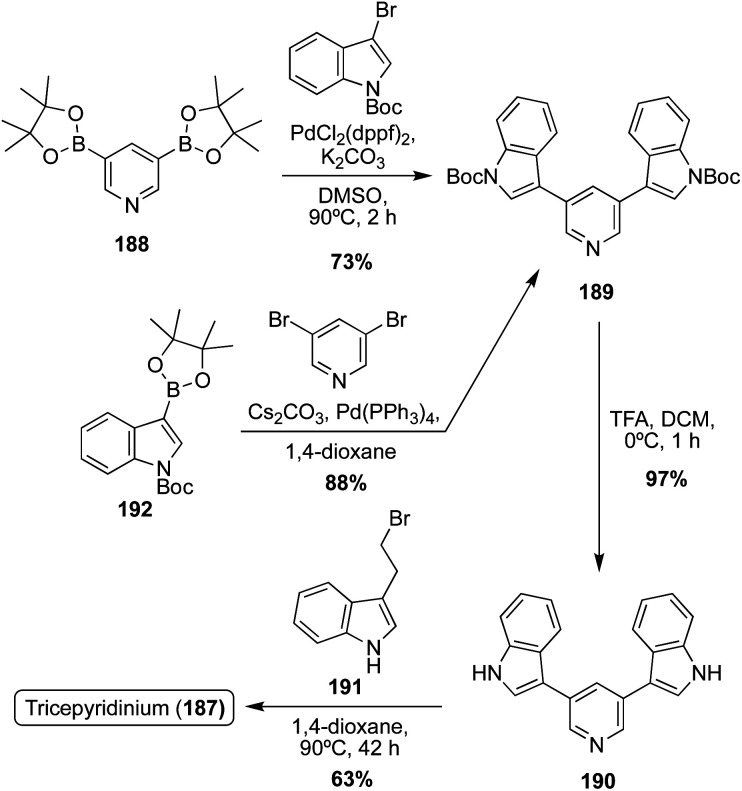
Total synthesis Tricepyridinium (187).

The first total synthesis of Tricepyridinium (187) was completed in 2017 by Abe, *et. al.* at the time of its isolation and structural elucidation to confirm its structure. As shown in Scheme 41, a bis-borylation of 3,5-dibromopyridine was completed to access intermediate 188, followed by a subsequent palladium-catalyzed double Suzuki coupling to install two of the indole moieties on the pyridine ring. Boc deprotection and late-stage alkylation of the pyridine nitrogen *via*191 to install the third indole moiety afforded Tricepyridinium (187) in high yields.^114^

In 2021, the total synthesis of Tricepyridinium (187) was completed *via* a similar approach as previously described. However, the major difference in this approach was the palladium catalyzed Suzuki coupling reaction, in which the electronics of the coupling partners were swapped. In this case, an indolic boronic ester (192) was coupled with 3,5-dibromopyridine, similar to the previously discussed approaches toward Scalaridine A (173). This approach led to a slightly higher yield of intermediate (189) of 88% compared to the previous 73% yield. Then, 189 was deprotected and acylated *via* alkyl bromide 191 to access Tricepyridinium (187) in 59% yield over two steps (Scheme 41).^115^

### 3.2 Non-aromatic N-heterocyclic linker moieties

#### 3.2.1. Tulongicin

**Fig. 16:**
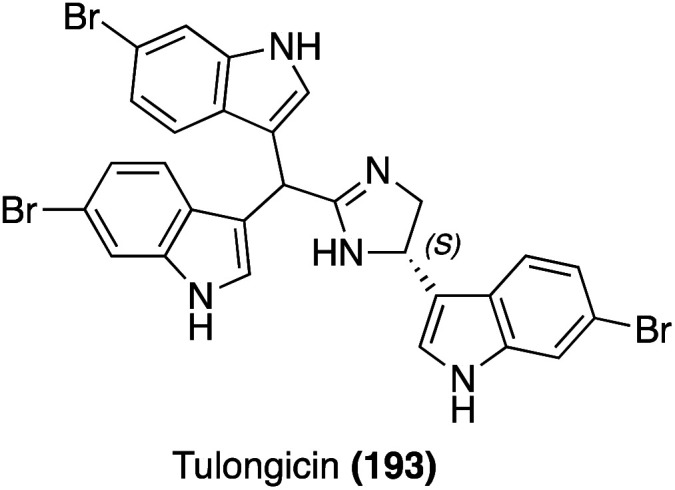
Chemical structure of Tulongicin (193).

Tulongicin (193) is a tri-indole alkaloid that was first isolated in 2017 by Liu *et. al.* from *Topsentia* sp., along with its previously discussed bis-indole alkaloid analogues, Dibromodeoxytopsentin (1h), Spongotine C (2c), and Dihydrospongotine C (3). Tulongicin (193) was structurally elucidated *via* spectroscopic methods. As shown in Fig. 16, Tulongicin (193) is comprised of an imidazoline core that links three brominated indole moieties. It is noteworthy to mention, 193 was the first marine alkaloid of its kind to contain the structurally complex bis-indole methane moiety connected to an imidazoline core. The absolute configuration of (4*S*)-193 was determined *via* comparison of experimental and calculated circular dichroism (CD) data (MPW1PW91/6-31G(d,p)). The only known biological activity of 193 is its strong antibacterial activity toward *S. aureus* (MIC 1.2 μg mL^−1^), its moderate anti-HIV activity (YU2: IC_50_ 3.9 μM, HxB2: IC_50_ 2.7 μM), and its lack of cytotoxicity in mammalian cells. It is also noteworthy to mention that Tulongicin (193) has yet to be accessed *via* total synthesis.^116^

#### 3.2.2. Araiosamines

**Fig. 17:**
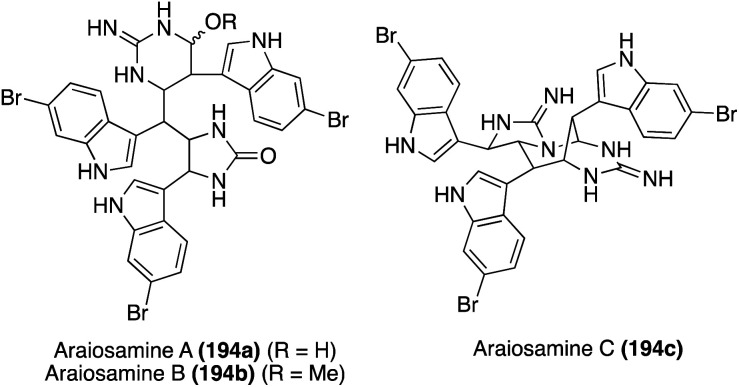
Chemical structures of Araiosamines A–C (194a–c).

The Araiosamines are arguably the most structurally complex of N-heterocyclic linked tris-indole alkaloid natural products that have been isolated to date. Araiosamines A–C (194a–c) were isolated in 2011 by Wei *et. al.* from the marine sponge *Clathria* (*Thalysias*) *araiosa*.^117^ As shown in Fig. 17, Araiosamines A and B (194a–b) contain three brominated indole moieties that are connected by cyclic guanidine and 2-imidazolinone linker moieties. The even more complex Araiosamine C (194c) contain three brominated indole moieties that are connected by a very complex fused and bridged cyclic guanidine core. These complex structures were elucidated *via* careful spectroscopic analysis. It is noteworthy to mention that the absolute configuration of these natural products is not known. The chemical structure of Araiosamine D falls outside the scope of this review.^117^ Interestingly, at the time of their isolation, Araiosamines A–C (194a–c) were not reported to exhibit any significant biological activity. However, several years later when some of these natural products were accessed *via* total synthesis, the Araiosamines were reported to display significant antibacterial activity against Gram-positive and Gram-negative bacteria, such as *S. aureus* and *E. coli*.^118^

**Scheme 42:**
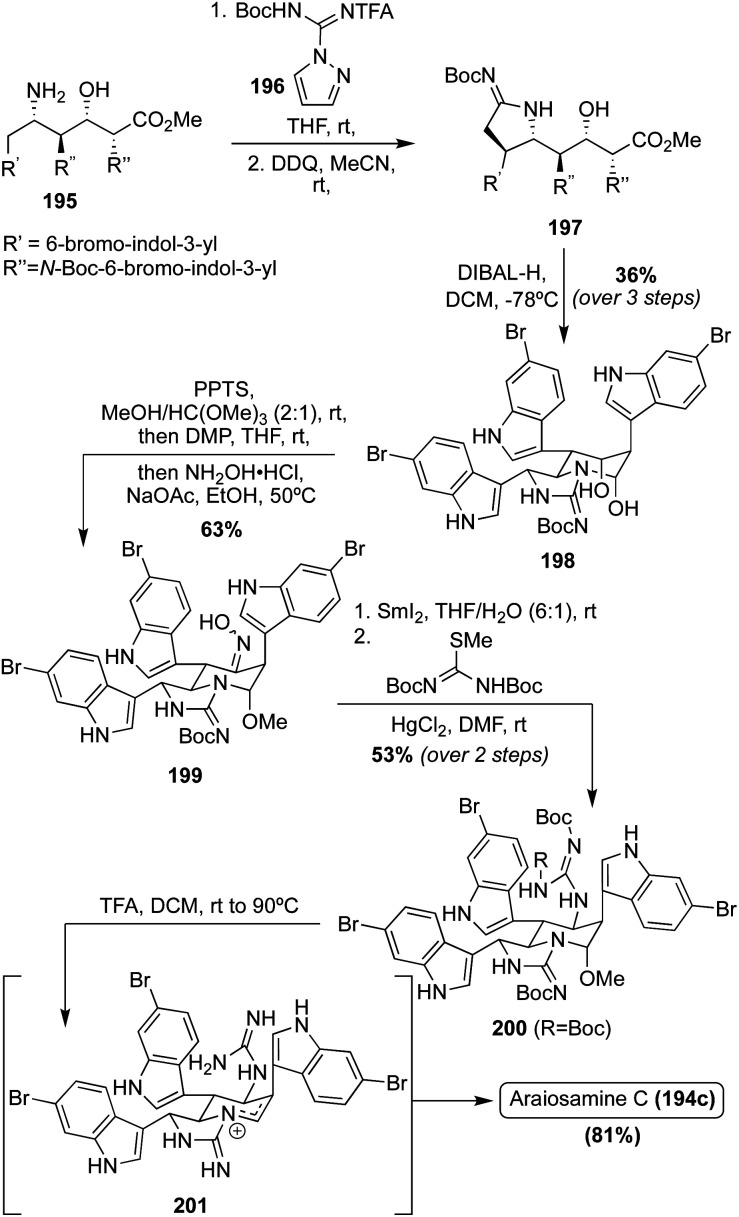
First total synthesis of Araiosamine C (194c).

Of these Araiosamine natural products, Araiosamine C (194c) is the only one to have been accessed *via* total synthesis. In 2016, Baran, *et. al.* completed the first total synthesis *via* key guanidine installation and selective C–H functionalization steps driving toward a biomimetic final step to construct the complex core of 194c. As is shown in Scheme 42, the first total synthesis of Araiosamine C (194c) began with formation of 195, followed by deprotection and subsequent guanidinylation. Many guanidinylation reagents that were screened led to no reaction and it was hypothesized that the TFA group would impart adequate reactivity for the guanidinylation reagent to react with hindered or electron deficient amines. Reduction of the ester and subsequent cyclization led to 198 in 36% yield over three steps. The oxime intermediate (199) was then synthesized and subsequently reduced to the primary amine intermediate *via* SmI_2_. The reduction of the oxime (199) was very challenging because the oxime moiety is sterically encumbered by two adjacent indole groups and other functionalities such as the *N*,*O*-acetal or three aryl bromides are likely more prone toward reduction. After reaction of the resulting amine with *N*,*N*-di-Boc-*S*-methylisothiourea, the guanidine intermediate (200) was accessed in 53% over two steps.^118^ As shown in Scheme 42, after deprotection and elimination of the methoxy group *via* acidic conditions and high temperatures, 201 was setup for the pivotal cyclization of the guanidine nitrogen and the fused cyclic imine/enamine to form the characteristic bridgehead of 194c. After this sequence, Araiosamine C (194c) was accessed in a very high 81% yield.^118^

**Scheme 43:**
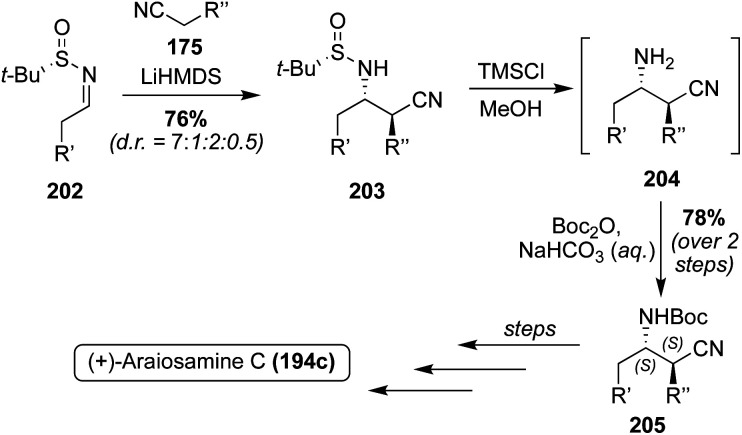
Enantioselective total synthesis of Araiosamine C (194c).

Since the absolute configuration of Araiosamine C (194c) was not known, the researchers adapted the previously discussed racemic path to 194c to gain access to a stereoselective synthesis. As shown in Scheme 43, this was done by utilizing Ellman's Auxiliary to synthesize the optically active 203 in 76% yield as the favored diastereomer (d.r. = 7 : 1 : 2 : 0.5). This stereochemistry was then retained through to 205, and using the same methodology as was previously discussed, the optically active (+)-Araiosamine C (194c) was synthesized, which was found to match the naturally isolated natural product based on optical rotation data.^118^ It is noteworthy to mention that Araiosamines A and B (194a–b) have yet to be accessed *via* total synthesis.

## 4. Conclusions

To conclude, the examples discussed in this review summarize the wide range of bis- and tris-indole alkaloids containing N-heterocyclic linker moieties that have been identified and isolated in recent years. These molecules exhibit potent biological activities, such as antibacterial, antiviral, cytotoxic, and anti-inflammatory activities. Natural products of this kind seem to share significant antibacterial activity overall. This could prove to be very advantageous in the future, as antibiotic resistance continues to be a major global issue. However, many of these previously discussed natural products have yet to be accessed *via* total synthesis, preventing their detailed biological evaluations. Development of synthetic methodologies to access these natural products and explore their promising biological activities will likely be of significant interest to the field in the future. It is also likely that the class of bis- and tris-indole natural products containing N-heterocyclic linker moieties will continue to expand as additional novel alkaloids are isolated.

## 5. Author contributions

Kyra R. Dvorak: conceptualization, writing original draft, writing, reviewing, and editing; Jetze, J. Tepe: supervision, reviewing, and editing.

## 6. Conflicts of interest

There are no conflicts to declare.
